# Zika virus: mapping and reprogramming the entry

**DOI:** 10.1186/s12964-019-0349-z

**Published:** 2019-05-03

**Authors:** Katarzyna Owczarek, Yuliya Chykunova, Christian Jassoy, Beata Maksym, Zenon Rajfur, Krzysztof Pyrc

**Affiliations:** 1 Virogenetics Laboratory of Virology, Malopolska Centre of Biotechnology, Jagiellonian University, Gronostajowa,7a 2.25, 30–387 Krakow, Poland; 2 Microbiology Department, Faculty of Biochemistry, Biophysics and Biotechnology, Jagiellonian University, Gronostajowa 7, 30-387 Krakow, Poland; 3 Institute for Virology, University Clinics and Medical Faculty, University of Leipzig, Leipzig, Germany; 4 Department of Pharmacology, School of Medicine with the Division of Dentistry in Zabrze, Medical University of Silesia in Katowice, Zabrze, Poland; 5 Institute of Physics, Faculty of Physics, Astronomy and Applied Computer Sciences, Jagiellonian University, Lojasiewicza 11, 30-348 Krakow, Poland

**Keywords:** ZIKV, Trafficking, Ammonium chloride, Bafilomycin A1, Endocytosis, Recycling, Furin

## Abstract

**Background:**

The *flaviviridae* family comprises single-stranded RNA viruses that enter cells via clathrin-mediated pH-dependent endocytosis. Although the initial events of the virus entry have been already identified, data regarding intracellular virus trafficking and delivery to the replication site are limited. The purpose of this study was to map the transport route of Zika virus and to identify the fusion site within the endosomal compartment.

**Methods:**

Tracking of viral particles in the cell was carried out with confocal microscopy. Immunostaining of two structural proteins of Zika virus enabled precise mapping of the route of the ribonucleocapsid and the envelope and, consequently, mapping the fusion site in the endosomal compartment. The results were verified using RNAi silencing and chemical inhibitors.

**Results:**

After endocytic internalization, Zika virus is trafficked through the endosomal compartment to fuse in late endosomes. Inhibition of endosome acidification using bafilomycin A1 hampers the infection, as the fusion is inhibited; instead, the virus is transported to late compartments where it undergoes proteolytic degradation. The degradation products are ejected from the cell via slow recycling vesicles. Surprisingly, NH_4_Cl, which is also believed to block endosome acidification, shows a very different mode of action. In the presence of this basic compound, the endocytic hub is reprogrammed. Zika virus-containing vesicles never reach the late stage, but are rapidly trafficked to the plasma membrane via a fast recycling pathway after the clathrin-mediated endocytosis.

Further, we also noted that, similarly as other members of the *flaviviridae* family, Zika virus undergoes furin- or furin-like-dependent activation during late steps of infection, while serine or cysteine proteases are not required for Zika virus maturation or entry.

**Conclusions:**

Zika virus fusion occurs in late endosomes and is pH-dependent. These results broaden our understanding of Zika virus intracellular trafficking and may in future allow for development of novel treatment strategies. Further, we identified a novel mode of action for agents commonly used in studies of virus entry.

Schematic representation of differences in ZIKV trafficking in the presence of Baf A1 and NH_4_Cl
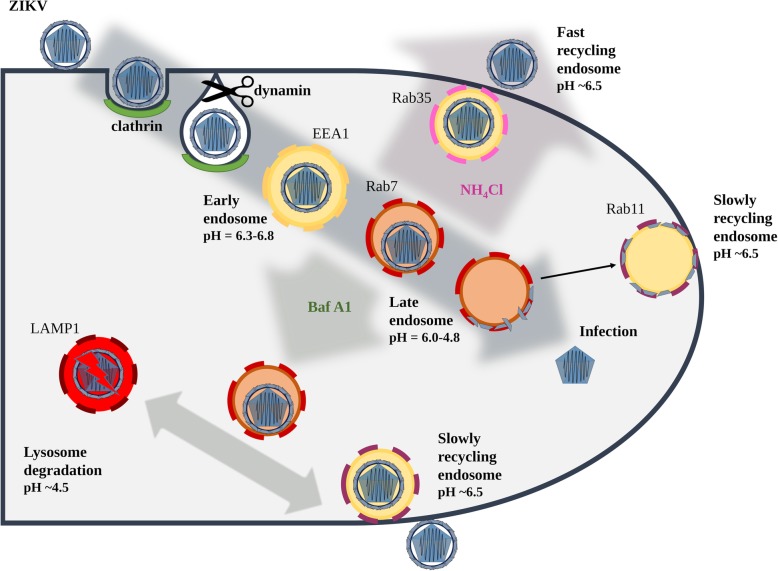

**Electronic supplementary material:**

The online version of this article (10.1186/s12964-019-0349-z) contains supplementary material, which is available to authorized users.

## Plain English summary

Zika virus is a mosquito-borne pathogen, which infects humans. Here, we present how Zika virus hijacks intracellular transport machinery to be delivered to the replication site. Tracking of single virions revealed that they follow clathrin-mediated endocytosis to fuse within late endosomes. Interestingly, we noted that two compounds commonly used to block endocytosis –bafilomycin A1 and NH_4_Cl - have a very different activity than previously anticipated. Bafilomycin A1 disables viral fusion by alteration of the endosome pH, while NH_4_Cl completely rewires the endosomal hub.

## Background

Zika virus (ZIKV) is a (+)ssRNA mosquito-borne flavivirus that infects humans [1]. Although it originates from Africa, it hit the spotlight almost 60 years after its first discovery, due to massive outbreaks in Micronesia, the South Pacific Islands, and South America [2–5]. It is estimated that, since identification of the first case in South America in May 2015, the total number of human cases on this continent has reached 1,300,000 [6]. The rapid spread and neuropathologic complications associated with ZIKV infection (microcephaly in newborns and Guillain-Barré syndrome in adults [7–9]), indicate an urgent need for research into the biology of the pathogen.

The initial events of ZIKV entry have been identified [10–12]; but data regarding its fate thereafter are limited. Upon attachment to a permissive cell, ZIKV crosses the plasma membrane via clathrin- and mucolipin-2-dependent endocytosis, accompanied by formation of LY6E tubules [13, 14]. The dependence of ZIKV on endocytosis has been confirmed in a variety of cell models [15].

Clathrin-mediated endocytosis (CME) is initiated by activation of receptor proteins, followed by recruitment of the AP2 adaptor complex, which induces assembly of the clathrin coat and formation of membrane niches of~ 100 nm [16]. As the invagination deepens, dynamin (GTPase), oligomerizes around the bud neck, cleaves it from the cell surface, and creates an intracellular vesicle [17, 18], which at first is translocated through the actin cortex and then trafficked along microtubules [19]. As the vesicle travels across the cell, it matures. First, the clathrin coat is removed; the uncoated vesicles may then fuse with each other or be delivered to the first (and major) sorting station, i.e., early endosomes. Vesicle trafficking is directed by small membrane GTPases belonging to the Rab family [20]. Early endosomes are characterized by the presence of early endosome antigen 1 (EEA1) and Rab5 proteins. During vesicle maturation, the pH gradually decreases due to the activity of proton pumps and fusion with other acidic vesicles. Early endosomes are moderately acidic (pH ∼ 6.3–6.8) [21], and their cargo can be sorted either for degradation via multivesicular bodies and late endosomes to lysosomes, or for recycling to the cell surface, exosomes, or the *trans*-Golgi network [22, 23]. Transport along the degradative pathway is associated with a gradual decrease in pH (from 6.0 to 4.8 in Rab7-positive late endosomes and further to 4.5 in lysosomes [24]). Lysosomes act as a storage site for hydrolases and other proteolytic enzymes; they are the final destination on this pathway. In recycling endosomes, the pH is maintained at ∼6.5 and vesicles may be targeted to the outer membrane by Rab35 via fast recycling endosomes (~ 5 min), or by Rab11 via slow recycling endosomes (15–30 min) [21]. Alternatively, cargo may be transported from multivesicular bodies to intraluminal vesicles, which may recycle to the cell surface via a Rab27a/b-mediated pathway, leading to release of cargo-loaded exosomes [25] (30–100 nm, often used by viruses during assembly and egress [26, 27]). Finally, at any point, cargos may enter the *trans*-Golgi network and follow the retrograde transport pathway guided by Rab9 [28].

The microenvironment within the vesicle during its travel is precisely controlled, and viruses usually fuse with the vesicular membrane at a certain time, i.e., when the pH, membrane composition, and activity of cellular proteases are optimal for fusion [22]. Virus dependence on an acidic environment is often treated as a requirement for endocytosis prior to fusion. Consequently, agents such as ammonium chloride (NH_4_Cl) or bafilomycin A1 (Baf A1), which increase intravesicular pH, are used to determine whether certain viruses are able to fuse to the cell surface or whether endocytic internalization is required.

Here, we complement and expand the knowledge about cell entry and intracellular trafficking of ZIKV. Tracking of single virions using confocal microscopy and separate labeling of the viral capsid and envelope proteins revealed that virions that enter cells via CME travel to late endosomal compartments and subsequently fuse with the membrane. Blocking endosome acidification using Baf A1 inhibited virus – cell fusion, leading to trafficking of virus either along the degradative pathway to the lysosomal compartments or its slow recycling to the cell surface. Similar results were expected for NH_4_Cl-treated cells; however, in this case, virions localized to the cell surface, suggesting a very different mechanism of action. Surprisingly, it appeared that NH_4_Cl “rewired” the endosomal hub and altered virus trafficking within the endocytic labyrinth.

## Materials and methods

### Virus and cells

African green monkey kidney (Vero) cells (ATCC number: CCL-81; RRID:CVCL_0059) were maintained at 37 °C under 5% CO_2_ in standard medium [Dulbecco-modified Eagle’s medium (DMEM, Thermofisher Scientific, Poland, Poland) supplemented with 3% heat-inactivated fetal bovine serum (FBS, Thermofisher Scientific, Poland), 100 U/ml penicillin and 100 μg/ml streptomycin (Immuniq, Poland)] with addition of 5 μg/ml ciprofloxacin.

ZIKV H/PF/2013 strain acquired from European Virus Archive [29] was propagated in Vero cells under standard medium. After 3 days of infection at 37 °C, virus-containing medium was collected and titrated. As a control, mock-infected Vero cells were subjected to the same procedure. Virus and mock aliquots were stored at − 80 °C. Virus titration was performed on confluent Vero cells on a 96-well plate, according to the Reed–Muench method [30].

### Inhibitors

Amantadine (1-adamantylaminean, AMTD; Sigma Aldrich, Poland) was used at 400 μM as clathrin-coated pit stabilizing agent [31]. Bafilomycin A1 [(3Z,5E,7R,8S,9S,11E,13E,15S,16R)-8-hydroxy-16-[(1S,2R,3S)-2-hydroxy-1-methyl-3-[(2R,4R,5S,6R)-tetrahydro-2,4-dihydroxy-5-methyl-6-(1-methylethyl)-2H-pyran-2-yl]butyl]-3,15-dimethoxy-5,7,9,11-tetramethyloxacyclohexadeca-3,5,11,13-tetraen-2-one, Baf A1; Sigma Aldrich, Poland] was used at 100 nM as a vacuolar type H^+^-ATPase inhibitor that hampers endosome acidification [32]. Camostat [4-[[4-[(aminoiminomethyl)amino]benzoyl]oxy] benzeneacetic acid 2-(dimethylamino)-2-oxoethyl ester methanesulfonate, cam; Sigma Aldrich, Poland] was used at 100 μM as a serine protease inhibitor [33]. Decanoyl-arg-val-lys-arg-chloromethylketone (CMK; Sigma Aldrich, Poland) was used at 50 μM as furin inhibitor [34]. Dynasore [3-hydroxynaphthalene-2-carboxylic acid (3,4-dihydroxybenzylidene) hydrazide, Dyn; Abcam, UK] was used at 100 μM as an inhibitor of the GTPase activity of dynamin [35]. Trans-epoxysuccinyl-L-leucylamido (4-guanidino) butane, L-trans-3-Carboxyoxiran-2-carbonyl-L-leucylagmatine, N-(trans-epoxysuccinyl)-L-leucine 4-guanidinobutylamide (E64; Sigma Aldrich, Poland) was used at 100 μM as a cysteine protease inhibitor [33]. MitMAB (tetradecyltrimethylammonium bromide, MM; Abcam, UK) was used at 20 μM as an inhibitor that targets dynamin-phospholipid interaction [36]. NH_4_Cl (Bioshop, Poland) was used at 50 mM as an intracellular alkalizing agent [37, 38]. PitStop 2 [N-[5-[4-bromobenzylidene]-4-oxo-4,5-dihydro-1,3-thiazol-2-yl] naphthalene-1-sulfonamide, PtS; Abcam, UK] was used at 50 μM as an inhibitor of ligand association with clathrin’s amino terminal domain [39].

### Immunostaining

Vero cells were seeded on glass slides in a cell culture plate and cultured in standard medium for two days at 37 °C. Upon experimental procedure, the cells were fixed with ice-cold 4% formaldehyde in PBS for 20 min at room temperature, washed with PBS and permeabilized with 0.5% TWEEN-20 for 10 min at room temperature. Afterwards, non-specific binding sites were blocked overnight at 4 °C with 5% BSA and slides were incubated for 2 h at room temperature with primary anti-ZIKV antibodies (specific to envelope protein (Merck Millipore, Poland) or capsid protein (kind gift from prof. Jassoy, Institut für Virologie, Leipzig, Germany) diluted 1:100 in 3% BSA in PBS. To visualize host cell proteins, slides were incubated with primary antibodies against clathrin, EEA1, Rab7, LAMP1, Rab11, Rab27 and Rab35 [goat anti-clathrin HC (RRID:AB_2083170), rabbit anti-EEA1 (RRID:AB_2277714) and rabbit anti-Rab7 (RRID:AB_2175483) polyclonal antibodies from Santa Cruz Biotechnology, Poland, rabbit anti-Rab11A (RRID:AB_2173458) polyclonal antibody from Proteintech, UK, rabbit anti-Rab27A monoclonal antibodies from Cell Signaling Technology, Poland, rabbit anti-Rab35 polyclonal antibody from Novus Biologicals, Poland, rabbit anti-LAMP1 (RRID:AB_2134611) polyclonal antibody from Thermofisher Scientific, Poland] diluted 1:100 in 3% BSA in PBS, together with anti-ZIKV antibodies. Next, the cells were incubated for another 1 h with Alexa Fluor 488-labeled goat anti-mouse IgG (RRID:AB_2534069, Thermofisher Scientific, Poland) or Alexa Fluor 488-labeled rabbit anti-mouse IgG (RRID:AB_2534106, Thermofisher Scientific, Poland) diluted 1:200 in 3% BSA in PBS. For staining of host cell proteins also Alexa Fluor 546 goat anti-rabbit secondary antibodies (RRID:AB_2534077, Thermofisher Scientific, Poland) or Alexa Fluor 546 donkey anti-goat secondary antibodies (RRID:AB_2534103, Thermofisher Scientific, Poland) diluted 1:200 were added to the mix. In experiments focused on siRNA silencing and inhibitors’ influence on virus internalization, the cell surface was labelled by Atto 633-phalloidin (Thermofisher Scientific, Poland) diluted 1:50 in PBS for 1 h at room temperature. Nuclei were stained with DAPI (RRID:AB_2629482, Thermofisher Scientific, Poland) diluted 1:10000 in PBS. At the end of immunostaining procedure slides were thoroughly washed with 0.5% TWEEN-20 in PBS. Stained slides were mounted in ProLong Diamond antifade medium (Thermofisher Scientific, Poland) and stored at 4 °C.

### Staining of living cells

Vero cells were seeded on 35 mm glass bottom dishes and cultured in standard medium for two days at 37 °C. Afterwards, the cells were washed with PBS and incubated in standard medium containing 50 nM LysoTracker™ Red DND-99 (Thermofisher Scientific, Poland) for 90 min at 37 °C to visualize acidic organelles. Next, the medium was discarded, and cells were overlaid with BafA1/NH_4_Cl-containing or control standard medium and observed for 30 min with a fluorescence microscope. The first images (“0 min”) were acquired in < 1 min upon treatment of the cells with the two above mentioned agents.

### Fluorescence and confocal microscopy

Images of living cells were acquired using EVOS FL Imaging System (Thermofisher Scientific, Poland) with 60× oil immersion lens. Images of fixed cells were taken under a ZEISS LSM 710 (version 8.1) confocal microscope with 40× oil immersion lens and ZEN 2012 SP1 (black edition, version 8.1.0.484). Image processing was performed with ImageJ FIJI (RRID:SCR_002285, National Institutes of Health, Bethesda, Maryland, USA). Co-localization parameters (Pearson’s and Manders’ coefficients) were calculated using JaCop plugin [40].

### Flow cytometry

Vero cells were seeded in a 6-well cell culture plate and cultured in standard medium for two days at 37 °C. Upon experiment, the cells were fixed, permeabilized, blocked and immunostained with primary antibodies specific to viral envelope protein (Merck Millipore, Poland) and secondary rabbit anti-mouse antibodies labeled with Alexa Fluor 488 (RRID:AB_2534106, Thermofisher Scientific, Poland), as indicated in *Immunostaining* section. Proportion of ZIKV-infected cells (corresponding to the median fluorescence of the analyzed cells population) was evaluated with flow cytometry using FACSCalibur (RRID:SCR_000401, Becton Dickinson, Poland). Cell Quest software (RRID:SCR_014489, Becton Dickinson, Poland) was used for data processing and analysis.

### Cell viability

Cells were seeded on 96-well plates and cultured in standard medium for two days at 37 °C. Afterwards, the cells were washed with PBS, overlaid with standard medium supplemented with inhibitor or control and further incubated for 3 days at 37 °C. Cell viability was examined using XTT Cell Viability Assay (Biological Industries, Poland), according to the manufacturer’s protocol. Briefly, the medium was discarded and 50 μl of fresh standard medium with 50 μl of the activated XTT solution was added to each well. After 2 h incubation at 37 °C, the supernatant was transferred onto a new, transparent 96-well plate and signal from formazan derivative of tetrazolium dye was read at λ = 490 nm using colorimeter (Tecan i-control Infinite 200 Microplate Reader, 1.5.14.0). The obtained results were further normalized to the control, where cell viability was set to 100%.

### Virus yield

Virus detection and quantification was performed using reverse transcription (RT) followed by quantitative real-time PCR (qPCR). Viral RNA was isolated from cell culture supernatant 3 days post-infection (p.i.) using Viral DNA / RNA Kit (A&A Biotechnology, Poland), while reverse transcription was carried out with High Capacity cDNA Reverse Transcription Kit (Thermofisher Scientific, Poland), according to manufacturers’ protocols. To assess virus yield, DNA standards were subjected to qPCR along with the cDNA acquired from the isolated samples. qPCR was performed using KAPA PROBE FAST qPCR Master Mix (Kapa Biosystem, Poland), ZIKV-specific primers (5′-TTG GTC ATG ATA CTG CTG ATT GC-3′ and 5′-CCT TCC ACA AAG TCC CTA TTG C-3′) and probe (5′-CGG CAT ACA GCA TCA GGT GCA TAG GAG-3′) labelled with FAM (6-carboxyfluorescein) and TAMRA (6-carboxytetramethylrhodamine). Rox was used as a reference dye. The signal was recorded and analysed using 7500 Fast Real-Time PCR System (Thermofisher Scientific, Poland).

### siRNA silencing

Control (scrambled) siRNA (sc-44,237) and pooled siRNAs targeting heavy chain of clathrin (sc-35,067) were obtained from Santa Cruz Biotechnology, Poland. siRNA specific to simian Rab35 mRNA (GenBank sequence ID: XM008004920.1) was designed and synthesized by Thermofisher Scientific, Poland.

Vero cells cultured for 1 day in antibiotic- and serum-depleted standard medium on a 6-well plate were transfected with appropriate siRNAs using Lipofectamine RNAiMAX (Thermofisher Scientific, Poland). The procedure was performed according to the manufacturer’s instructions and repeated 24 h later to enhance the silencing effect. The efficiency of the procedure was assessed by Western blotting 24 h later (at the same time as microscopic studies on virus subcellular localization).

### Western blot analysis

Cells were harvested in RIPA buffer (1 h, 4 °C; Thermofisher Scientific, Poland) supplemented with 0.5 M EDTA and protease inhibitors cocktail (cOmplete Tablets, Roche, Poland)]. Protein concentration was assessed with Pierce BCA Protein Assay Kit (Thermofisher Scientific, Poland), according to manufacturer’s protocol. Samples containing equal amounts of proteins were mixed with SDS-PAGE Sample Buffer (0.5 M Tris, pH 6.8, 10% SDS, 50 mg/ml DTT), denatured for 10 min at 95 °C and separated by SDS-PAGE electrophoresis. Subsequently, proteins were electrotransferred onto a PVDF membrane (1.5 h, 100 V; Amersham, Poland). The non-specific binding sites on the membrane were blocked for 1 h at room temperature with 10% milk (Bioshop) in Tris-buffered saline supplemented with 0.25% TWEEN-20 (Bioshop, Poland) (TBST) and incubated with primary antibodies specific to clathrin or Rab35 [rabbit anti-clathrin heavy chain polyclonal antibody, RRID:AB_10695306, Cell Signaling Technology, Poland; rabbit anti-Rab35 polyclonal antibody, Novus Biologicals, Poland] diluted 1:500 or 1:1000 (for clathrin and Rab35, respectively) in 3% BSA in TBST overnight at 4 °C and additionally for 1 h at room temperature; or with primary antibodies specific to GAPDH (rabbit anti-GAPDH antibodies, RRID:AB_561053, Cell Signaling Technology, Poland) diluted 1:5000 in 3% BSA in TBST for 1 h at room temperature. After being washed in TBST, the membrane was incubated with HRP-labelled anti-rabbit IgG antibody (RRID:AB_257896, Sigma Aldrich, Poland) diluted 1:20,000 in 3% BSA in TBST for 1 h at room temperature. Finally, the proteins were visualized with chemiluminescence, using the ECL system (Amersham, Poland).

### Co-localization assay

Vero cells were seeded in standard medium on glass slides in 12-well plates. After 2 days, 2 h prior to infection, cell culture medium was replaced with serum-depleted standard medium. Next, cells were cooled down to 4 °C, overlaid with 100 μl of non-diluted ZIKV stock (TCID_50_ ranging from 1000,000 to 10,000,000/ml, which approximately corresponds to MOI = 1.75–17.5 and 7 × 10^5^–7 × 10^6^ PFU/ml) and incubated for 30 min at 4 °C to synchronize cargo particles entry from the cell surface. Subsequently, after incubation at 37 °C (exact times indicated for each experiment) the cells were fixed, permeabilized, blocked and immunostained for viral and cellular proteins, as indicated in *Immunostaining* section.

### Virus inhibition assays

For visualization of virus subcellular localization during experiments with agents interfering with virus trafficking, Vero cells were cultured in standard medium on glass slides in 12-well plates for 2 days and pre-treated with a particular inhibitor 1 h prior to infection. Afterwards, the cells were cooled down to 4 °C, overlaid with 100 μl of non-diluted ZIKV stock (TCID_50_ ranging from 1000,000 to 10,000,000/ml, which approximately corresponds to MOI = 1.75–17.5 and 7 × 10^5^–7 × 10^6^ PFU/ml) in the presence of inhibitory agents and incubated for another 30 min at 4 °C to synchronize cargo internalization. Subsequently, the virus-overlaid cells were warmed up to 37 °C. At indicated for each experiment time points, cells were washed with PBS, fixed, permeabilized, blocked and immunostained for viral and actin cytoskeleton proteins as indicated in *Immunostaining* section. Identical procedure was carried out for visualization of virus particles in cells depleted of certain proteins with siRNAs.

To test the influence of compounds on virus adhesion, Vero cells cultured in standard medium in a 6-well plate for 2 days were cooled down to 4 °C, overlaid with 100 μl of ice-cold non-diluted ZIKV stock (TCID_50_ ranging from 1000,000 to 10,000,000/ml, which approximately corresponds to MOI = 1.75–17.5 and 7 × 10^5^–7 × 10^6^ PFU/ml) and incubated for another 2 h at 4 °C. Further, cells were rinsed twice with ice-cold PBS. The cells were fixed, permeabilized, blocked, immunostained and analyzed with flow cytometry, as described in *Flow cytometry* section.

For assessment of the inhibitors’ influence on viral replication, Vero cells were cultured in standard medium in 96-well plates for 2 days and pre-treated with a selected agent 1 h prior to infection. Virus at TCID_50_ of 800/ml (which approximately corresponds to MOI = 0.0014 and 550 PFU/ml) was overlaid on the cells in the presence of inhibitors and samples were incubated for 2 h at 37 °C. Wells were washed thrice with PBS and incubated at 37 °C in standard medium supplemented with inhibitors. 3 days p.i. culture supernatants were collected, viral RNA was isolated and its yield was quantified with RT-qPCR. Whether the procedure was modified, it is described in the results section.

### Statistical analyses

Each experiment was performed at least twice in triplicate. Chart bars represent mean ± SD. The significance of differences between compared groups was determined by Single-Factor Analysis of Variance (ANOVA); *p* values < 0.05 were considered significant.

## Results

### ZIKV enters Vero cells via clathrin-dependent endocytosis

**Fig. 1:**
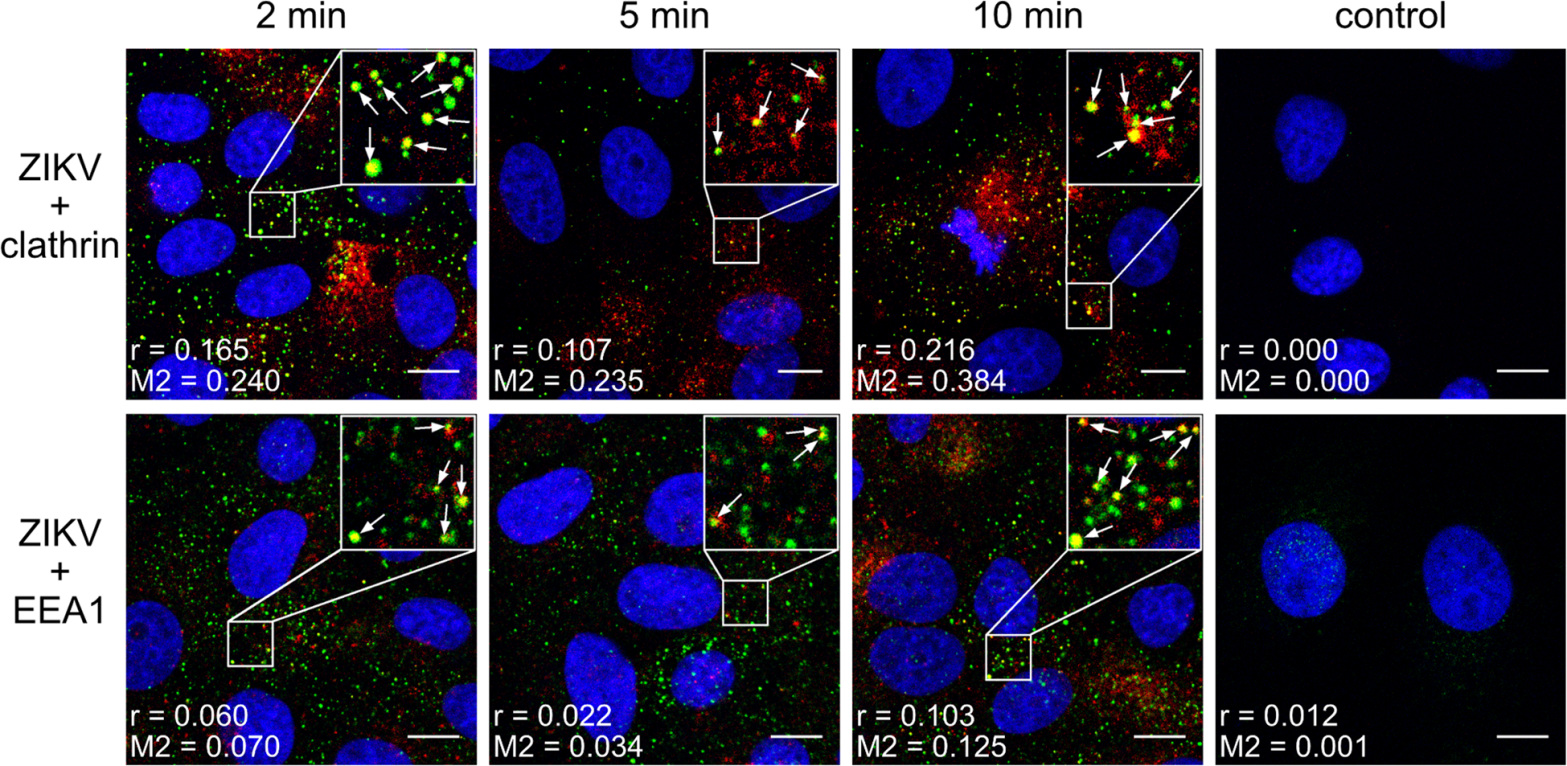
Co-localization between ZIKV and clathrin-dependent endocytosis markers. Confocal images of Vero cells showing co-localization of ZIKV envelope protein and clathrin (upper) or EEA1 (lower) at different time points p.i. Respective time points are indicated at the top of each column. Control denotes mock-infected cells, stained with anti-ZIKV envelope antibodies and rabbit isotype antibodies (control for staining of cellular proteins). The virus is visualized in green, clathrin and EEA1 are shown in red, and nuclei are presented in blue. Scale bar = 10 μm. Co-localization parameters: r – Pearson’s coefficient; M2 - Manders’ coefficient M2 (the virus overlapping with clathrin/EEA1)

First, we asked whether ZIKV enters Vero cells via CME, as reported for other in vitro systems. We examined co-localization of virions and cellular proteins (clathrin, caveolin, endophilin, EEA1) at several time points post-infection (p.i.). Co-localization of ZIKV with clathrin was observed at 2–10 min p.i. and coincided with co-localization of virus with the early endosomal marker EEA1 (Fig. 1). No co-localization with caveolin or endophilin was observed (data not shown).

**Fig. 2:**
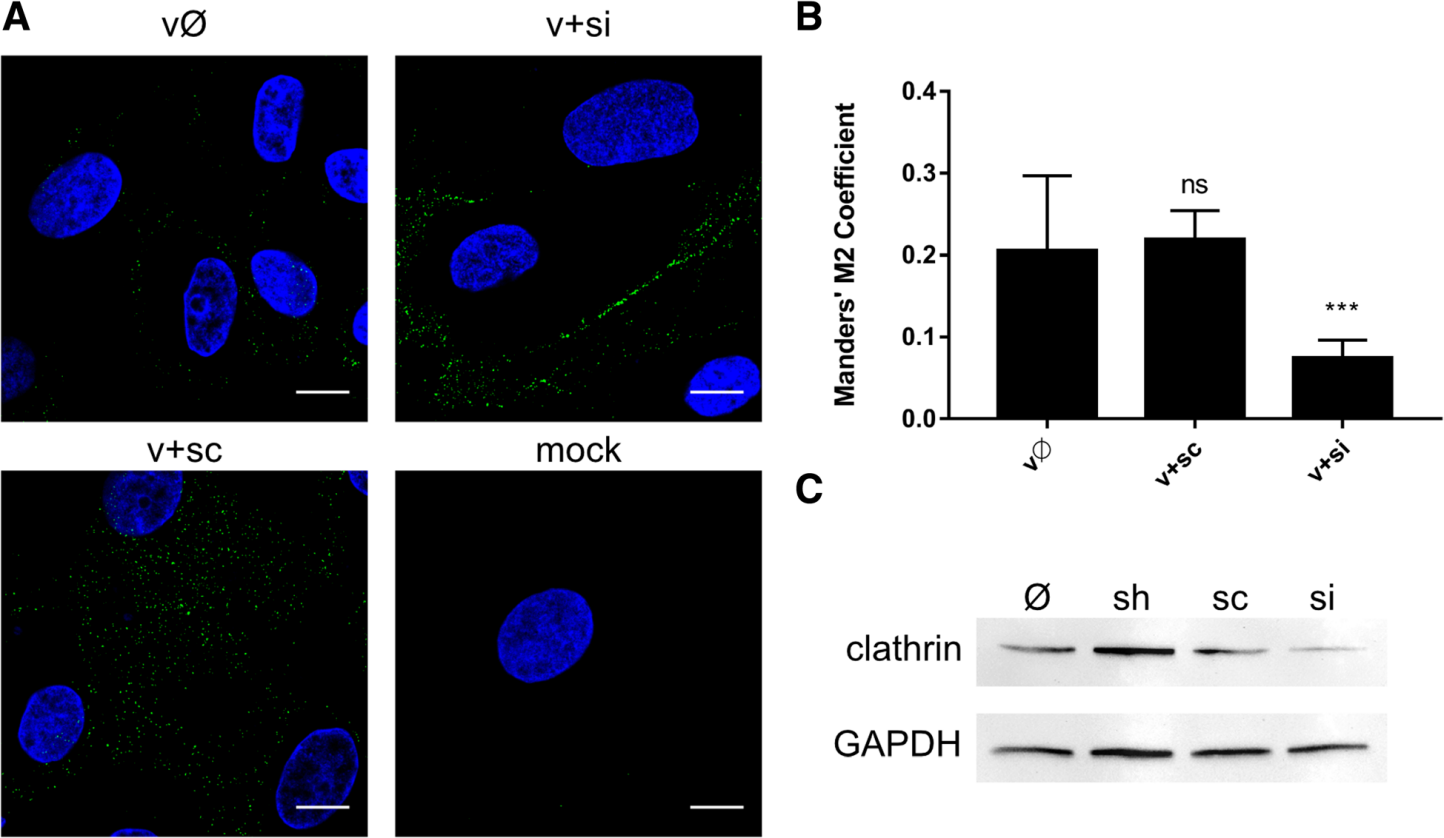
Inhibition of ZIKV entry to clathrin-depleted cells. (**a**) Confocal analysis of ZIKV localization 10 min p.i. in Vero cells. vØ – ZIKV-infected, non-transfected cells; v + si – ZIKV-infected, clathrin-specific siRNA transfected cells; v + sc – ZIKV-infected, scrambled siRNA transfected cells; v + sh – ZIKV-infected, sham transfected cells; mock – mock-infected, non-transfected cells. ZIKV envelope protein is visualized in green and nuclei are shown in blue. Scale bar = 10 μm. (**b**) Co-localization between ZIKV envelope and clathrin presented as Manders’ coefficient M2 for control and clathrin-depleted Vero cells inoculated with ZIKV. The data is presented as mean ± SD. To determine the significance of differences between compared groups, single-factor analysis of variance (ANOVA) was applied. *P* values < 0.05 were considered significant. One asterisk (*) identifies adjusted P values between 0.01 and 0.05, two asterisks (**) identify adjusted *P* values between 0.01 and 0.001, three asterisks (***) identify adjusted *P* values between 0.001 and 0.0001. (**c**) Western blot analysis of the efficiency of siRNA-dependent clathrin silencing (clathrin expression in Vero cells compared to GAPDH expression in these cells). M – BlueStar prestained protein marker; Ø – normal non-transfected Vero cells

As co-localization rates were not very high (Pearson’s coefficient ranging from 0.103 to 0.216 and Manders’ coefficients up to 0.384), we carried out a complementary study to validate the results. Subcellular localization of ZIKV in Vero cells in which clathrin expression was transiently silenced was examined. In this model virions were retained on the surface of clathrin-depleted cells even at 10 min p.i.; by contrast, control cells showed normal expression of clathrin and were permissive for ZIKV entry (Fig. 2).

**Fig. 3:**
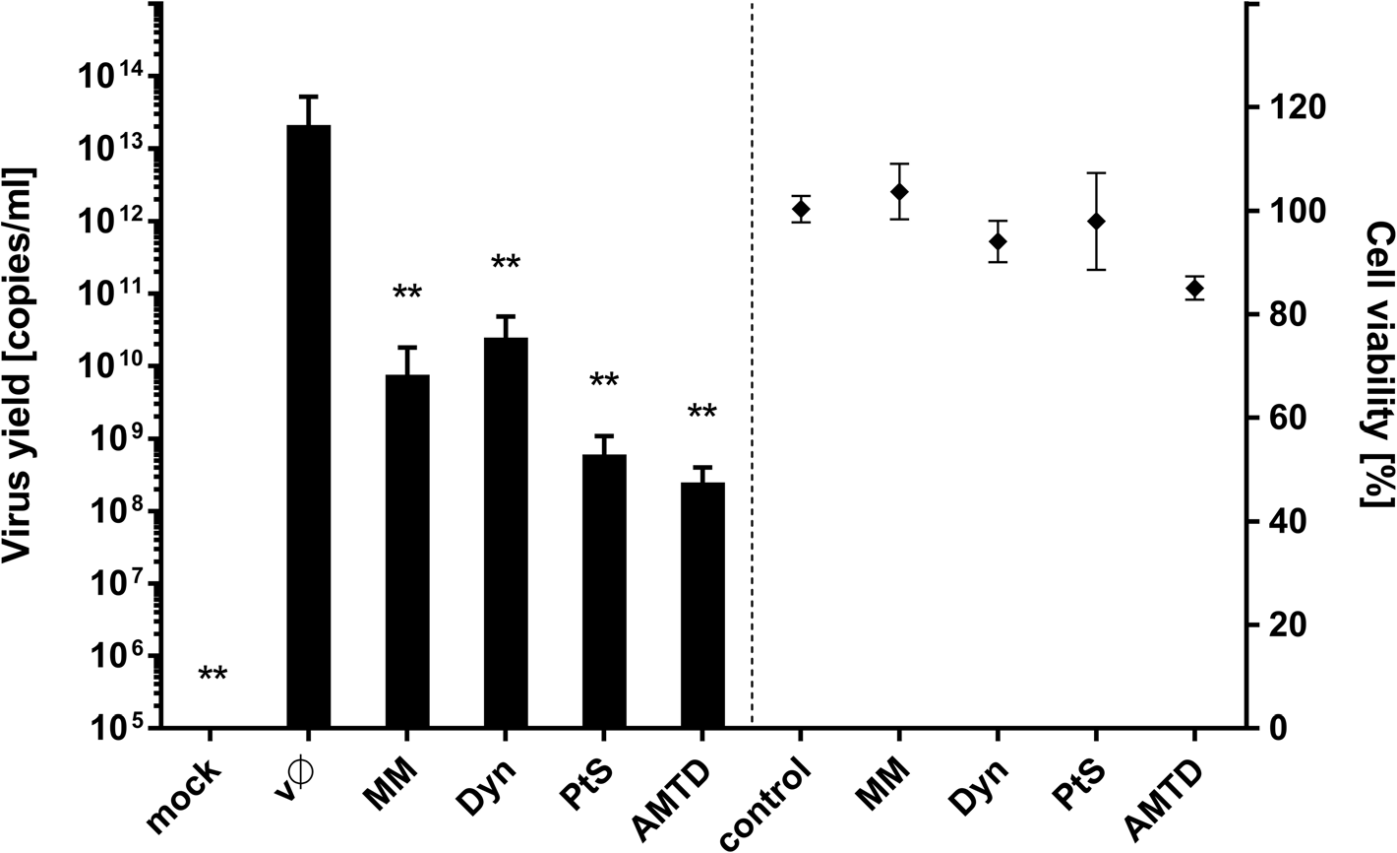
Inhibition of ZIKV infection in Vero cells by chemical agents blocking clathrin-mediated endocytosis. Vero cells pre-treated with CME inhibitors were infected with ZIKV and viral yield was assessed 3 days p.i. Virus yield (RT-qPCR) is presented on the left side of the graph, while on the right side toxicity of the compounds is visualized (XTT assay). mock – mock-infected cells; vØ – ZIKV-infected cells; AMTD – 400 μM amantadine, Dyn – 100 μM dynasore, MM – 20 μM MitMab, PtS – 50 μM PitStop; control – inhibitor-untreated, non-infected cells. The data is presented as mean ± SD. To determine the significance of differences between compared groups, single-factor analysis of variance (ANOVA) was applied. *P* values < 0.05 were considered significant. One asterisk (*) identifies adjusted P values between 0.01 and 0.05, two asterisks (**) identify adjusted P values between 0.01 and 0.001, three asterisks (***) identify adjusted P values between 0.001 and 0.0001

To prove that clathrin-dependent endocytosis is the main route of ZIKV internalization in Vero cells, we examined the effects of CME inhibitors on virus replication. We selected compounds to target multiple steps of the vesicle formation process. These included PitStop (PtS), which inhibits association between ligands with the terminal domain of clathrin [39]; amantadine (AMTD), which stabilizes clathrin-coated pits [31]; MitMAB (MM), which targets the dynamin-phospholipid interaction [36]; and dynasore (Dyn), which inhibits the GTPase activity of dynamin and therefore impairs loss of loaded vesicle from the cell surface [35]. We assessed the impact of each inhibitor on ZIKV infection by measuring the viral yield released from infected cells into the medium at 3 days p.i. All compounds reduced the ZIKV yield significantly (Fig. 3), suggesting that ZIKV enters the Vero cells via the clathrin-dependent route, as reported for human cells [10].

### ZIKV fusion occurs in late endosomal compartment

**Fig. 4:**
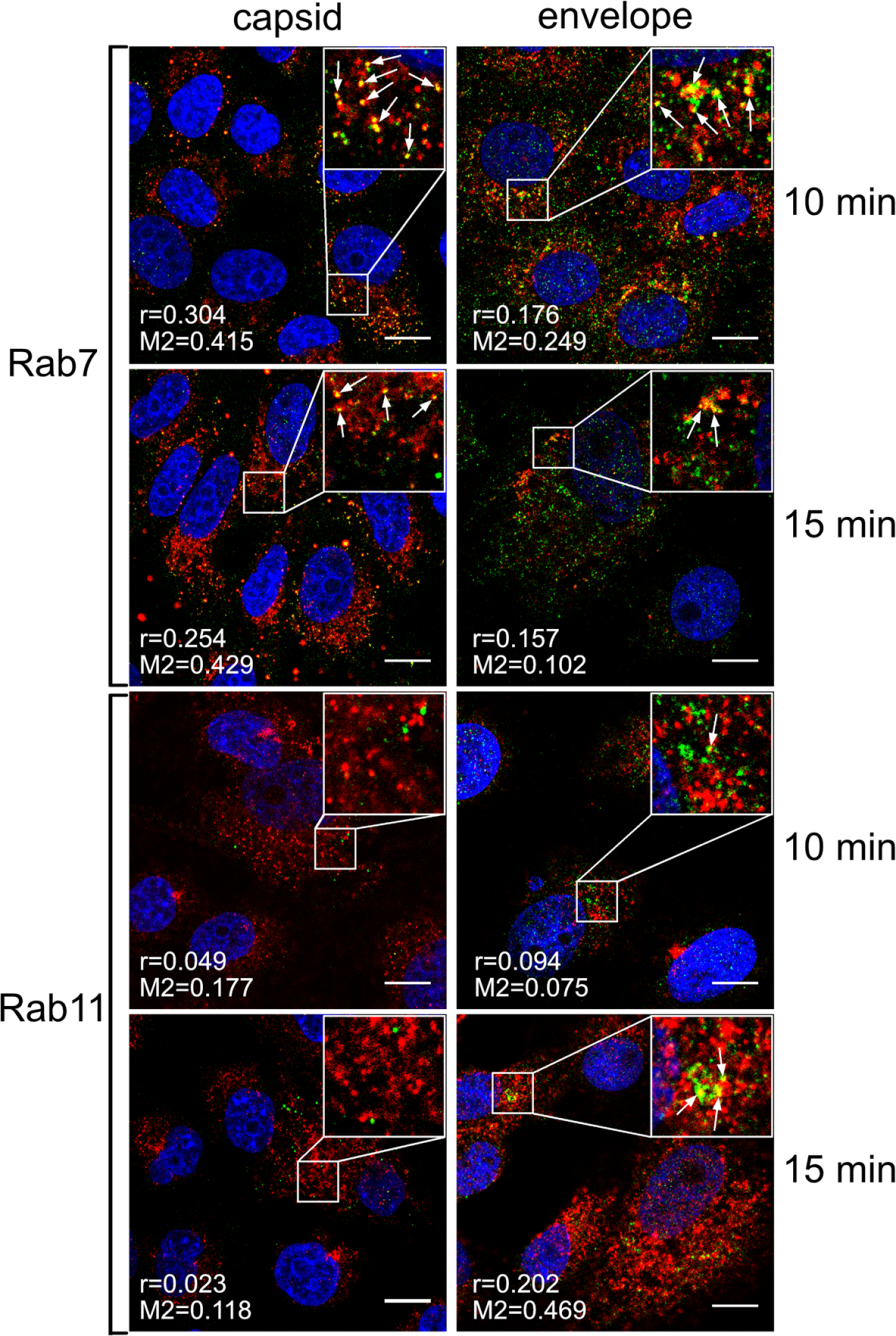
Co-localization of ZIKV with Rab7 and Rab11 marker proteins 10–15 min p.i.

Once in the endosomal hub, ZIKV has a plethora of pathways by which it can reach the site of fusion with the host cell membrane. Conditions within endosomal compartments differ with respect to lipid/protein content and pH. To find out how the virus is trafficked to reach the site of fusion, using confocal microscopy we tracked two ZIKV structural proteins, the capsid protein (virus core) and the membrane-bound envelope protein. Co-localization of both viral proteins with cellular proteins (Rab7 in late endosomes, Rab11 in slow recycling endosomes, and LAMP1 in lysosomes) was analyzed at 5–20 min p.i. (full set of images is available in Additional file 1: Figure S1 and Additional file 2: Figure S2). The most evident co-localization of the ZIKV capsid was found with Rab7, peaking at 10–15 min p.i. (Fig. 4). However, the envelope protein showed increased co-localization with both Rab7 and Rab11-positive structures at 15 min p.i. (Fig. 4), suggesting that slowly recycling endosomes may carry viral proteins to the cell surface upon delivery of RNA to the cytoplasm from late endosomes. Finally, no co-localization with LAMP1 was found (Additional file 1: Figure S1 and Additional file 2: Figure S2).

### Role of cellular proteases during ZIKV infection

**Fig. 5:**
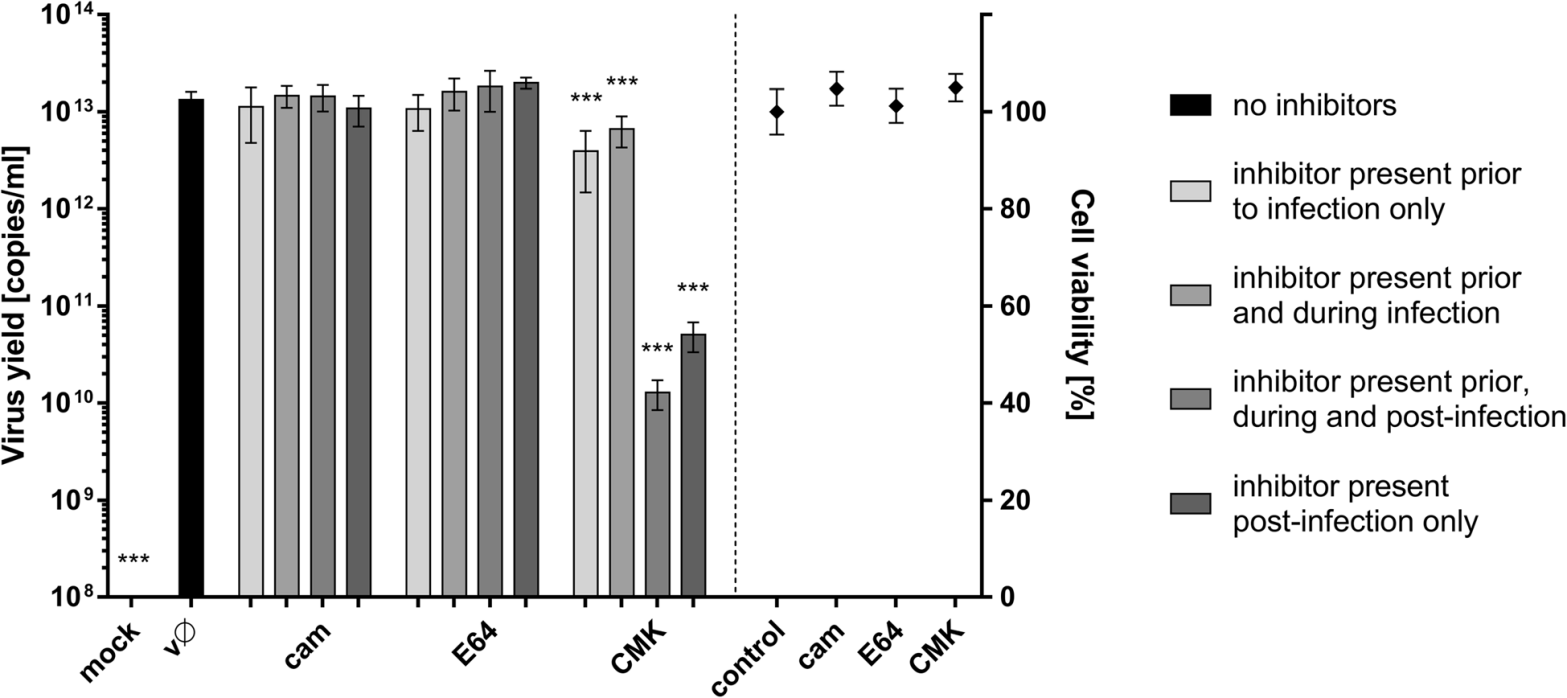
Cellular proteases and ZIKV. Vero cells were treated with proteases inhibitors (as outlined in the figure) and virus yield 3 days p.i. was assessed using RT-qPCR (left side of the graph); cytotoxity of inhibitors is presented on the right side of the graph (XTT assay). mock – mock infected cells; vØ – ZIKV- infected cells; cam – 100 μM serine protease inhibitor camostat; E64–100 μM cysteine protease inhibitor E64; CMK – 50 μM furin inhibitor CMK; control – inhibitor-untreated, non-infected cells. The data is presented as mean ± SD. To determine the significance of differences between compared groups, single-factor analysis of variance (ANOVA) was applied. P values < 0.05 were considered significant. One asterisk (*) identifies adjusted P values between 0.01 and 0.05, two asterisks (**) identify adjusted P values between 0.01 and 0.001, three asterisks (***) identify adjusted *P* values between 0.001 and 0.0001

Upon identification of the fusion site, we next identified host factors taking part in this process. Virus escape from the endosomal compartment usually occurs upon activation of viral fusion proteins, which may be triggered by environmental conditions or/and host proteases [41]. Therefore, we examined the importance of different host cell proteases. Vero cells were treated either with a furin inhibitor (decanoyl-arg-val-lys-arg-chloromethylketone [CMK]), a serine protease inhibitor (camostat [cam]), or a cysteine protease inhibitor (E64) under four different conditions: (1) 1 h prior to infection; (2) 1 h prior to infection and for 2 h during infection; (3) 1 h prior to infection and for 72 h p.i.; and (4) 2 h p.i. to 72 h p.i.. Culture supernatants were collected at 72 h p.i., and viral RNA was isolated and quantified by RT-qPCR. As shown in Fig. 5, infection was not affected by serine and cysteine protease inhibitors; however, the furin inhibitor led to a significant decrease in virus yield when administered p.i., suggesting that furin or furin-like enzymes play an important role during ZIKV replication, assembly or egress.

### Agents that increase endosomal pH hamper ZIKV entry and infection

**Fig. 6:**
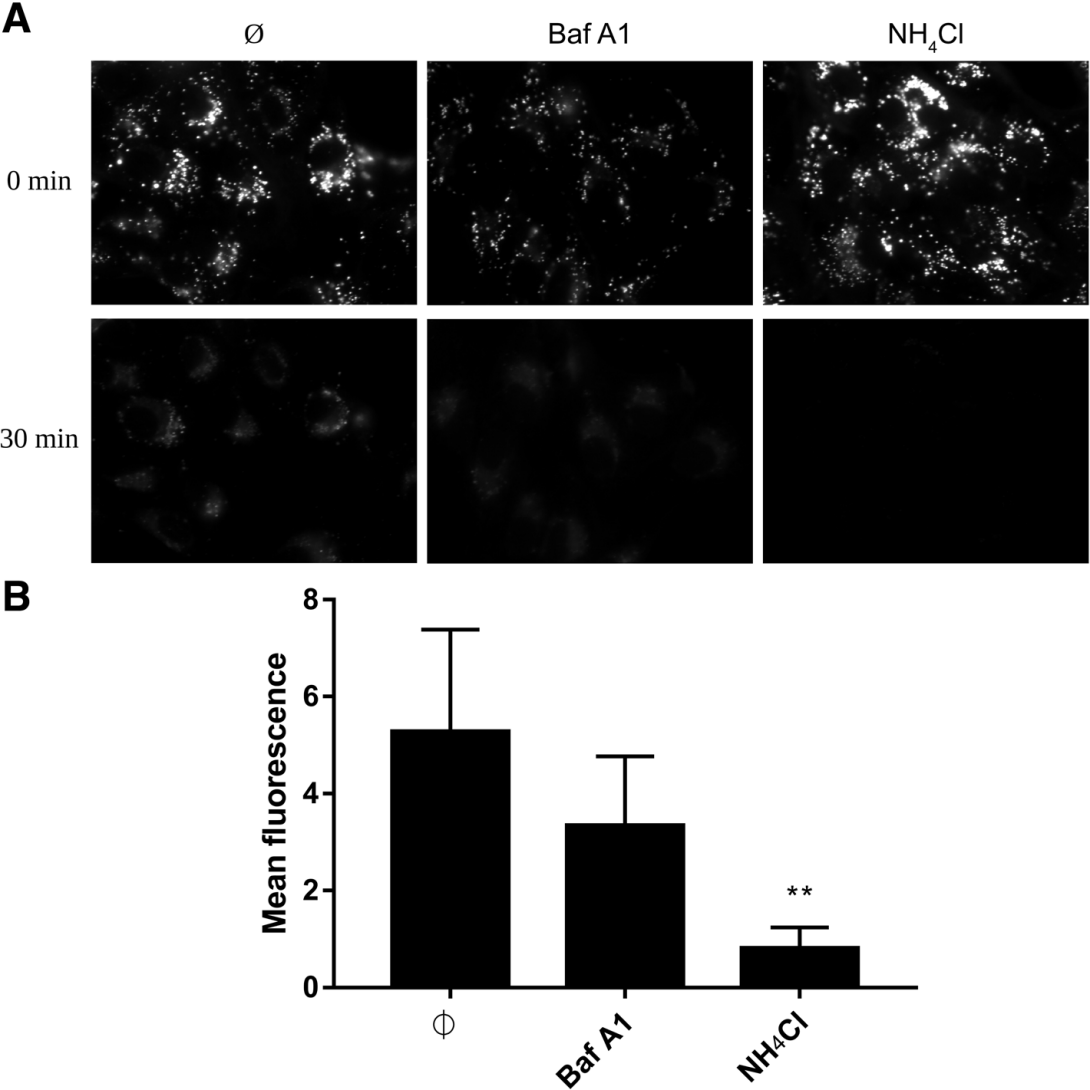
Endosome acidification in living cells treated with Baf A1 and NH_4_Cl. Vero cells were incubated with LysoTracker™ Red DND-99 for 90 min at 37 °C and overlaid with BafA1, NH_4_Cl or control medium (Ø) and signal was recorded for 30 min with a fluorescence microscope. (**a**) Images acquired at the beginning (0 min) and by the end (30 min) of incubation. (**b**) Mean fluorescence of the LysoTracker™ Red DND-99 based on the set of xy images collected every 10 s in a 30 min period. The data is presented as mean ± SD. To determine the significance of differences between compared groups, single-factor analysis of variance (ANOVA) was applied. *P* values < 0.05 were considered significant. One asterisk (*) identifies adjusted P values between 0.01 and 0.05, two asterisks (**) identify adjusted P values between 0.01 and 0.001, three asterisks (***) identify adjusted P values between 0.001 and 0.0001

**Fig. 7:**
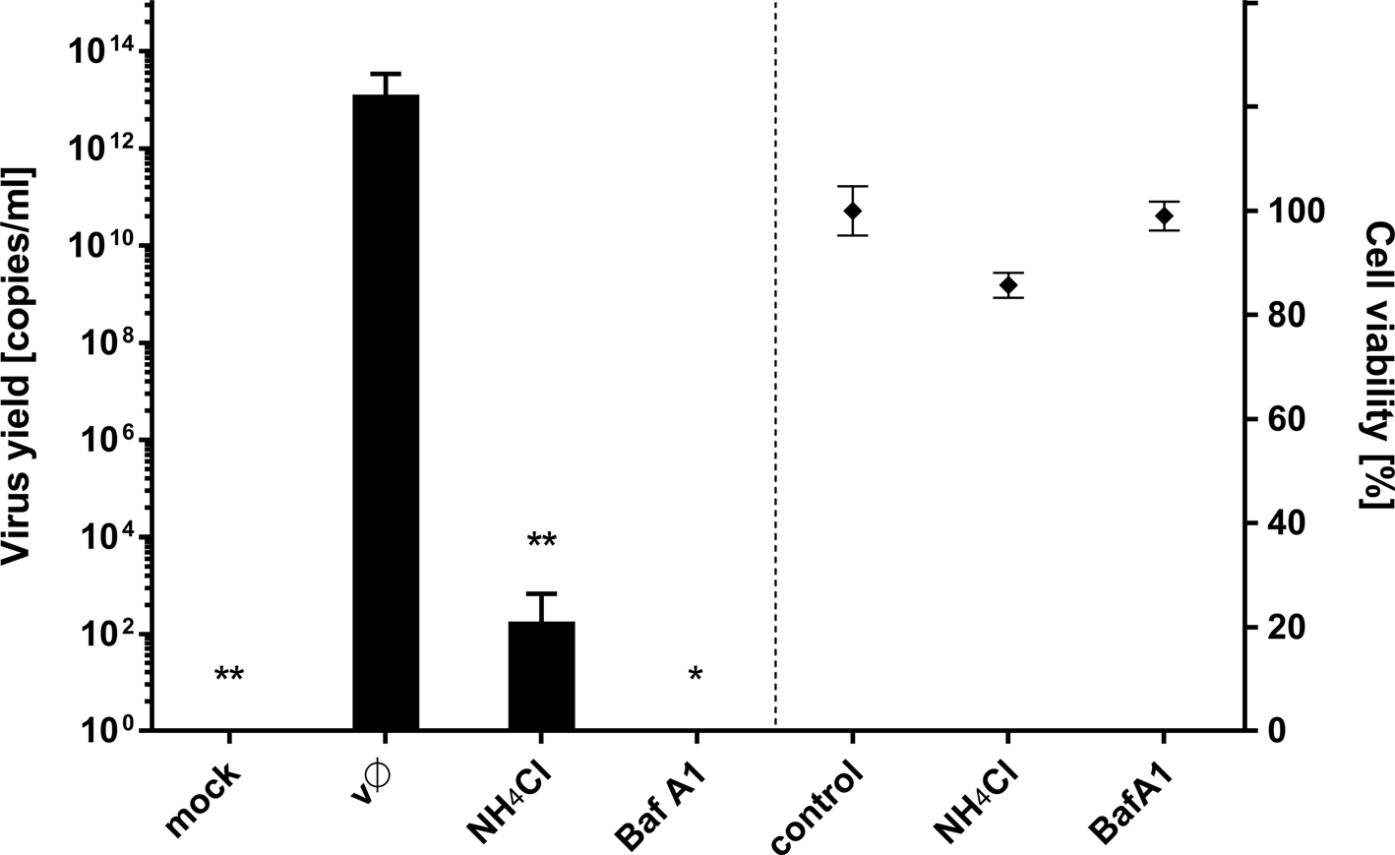
Baf A1 and NH_4_Cl inhibit ZIKV infection. Vero cells pre-treated with intracellular agents hampering endosome acidification were infected with ZIKV and viral yield was assessed at 3 days p.i. RT-qPCR results are presented on the left side of the graph. Right side of the graph shows viability of cells, as determined by an XTT assay. Mock – mock infected cells; vØ – ZIKV- infected cells; NH_4_Cl – 50 mM NH_4_Cl; BafA1–100 nM bafilomycin A1; control – inhibitor-untreated, non-infected cells. The data is presented as mean ± SD. To determine the significance of differences between compared groups, single-factor analysis of variance (ANOVA) was applied. P values < 0.05 were considered significant. One asterisk (*) identifies adjusted P values between 0.01 and 0.05, two asterisks (**) identify adjusted P values between 0.01 and 0.001, three asterisks (***) identify adjusted P values between 0.001 and 0.0001

**Fig. 8:**
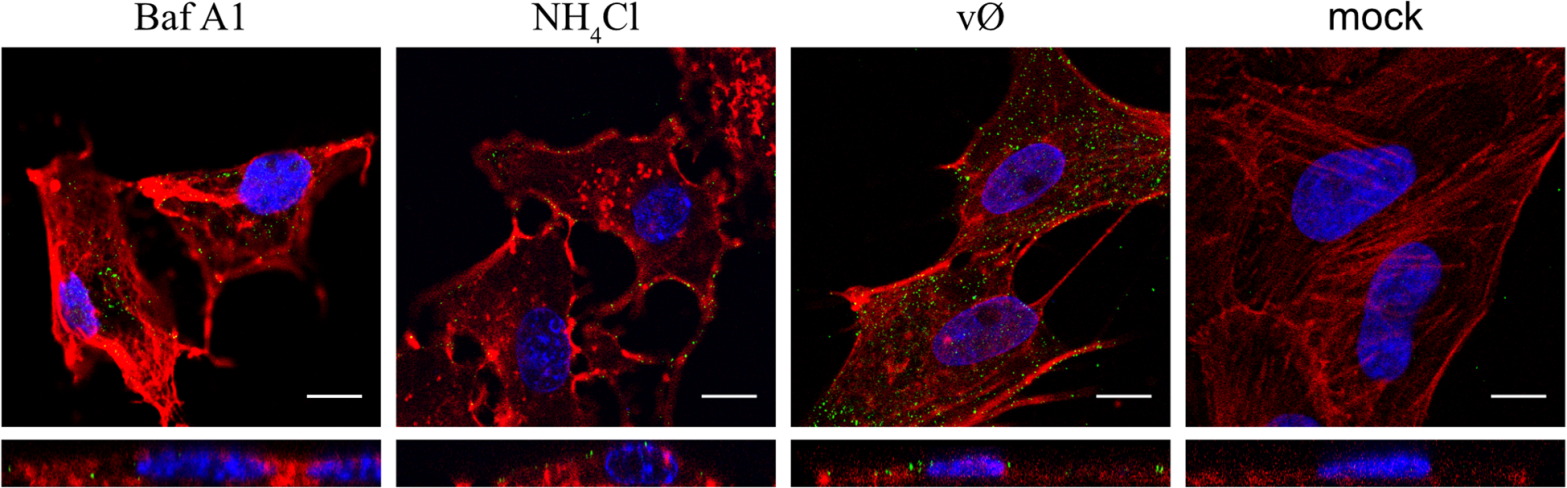
Increased number of virions trapped in the endosomal hub in cells treated with Baf A1, but not NH_4_Cl. Confocal analysis of ZIKV entry to Vero cells in the presence of intracellular agents hampering endosome acidification. Following 1 h pre-treatment with 100 nM Baf A1 or 50 mM NH_4_Cl the cells were infected with ZIKV and fixed 40 min p.i. Baf A1 - ZIKV-infected Baf A1-treated cells; NH_4_Cl - ZIKV-infected NH_4_Cl -treated cells; vØ - ZIKV-infected inhibitor-untreated cells; mock – mock-infected, inhibitor-untreated cells. ZIKV envelope protein is visualized in green, actin cytoskeleton stained in red to show the cell boundaries and nuclei are shown in blue. Scale bar = 10 μm. Upper panel – *xy* projections; bottom panel – *xz* projections

We know that for some flaviviruses cell entry is sensitive to pH changes; therefore, we used two compounds that increase intravesicular pH (Fig. 6) to check the pH dependence of ZIKV entry. Vero cells were treated with NH_4_Cl or Baf A1 1 h prior to infection. Next, cells were infected with ZIKV in the presence of NH_4_Cl or Baf A1 for 3 days at 37 °C. RT-qPCR analysis revealed strong inhibition of infection (Fig. 7). Next, we used confocal microscopy to test whether the compounds indeed inhibit virus entry. Cells were treated with either of the inhibitors for 1 h and then incubated with ZIKV for 40 min in the presence of the inhibitors. Confocal microscopy revealed that viral particles in Baf A1-treated cells were visible in the cytoplasm, probably trapped in the endosomal hub and unable to undergo fusion (Fig. 8). Interestingly, we observed a different intracellular virus distribution in cells treated with NH_4_Cl. Only a small number of ZIKV virions was visible in the cytoplasm, while ZIKV particles localized mainly to the cell surface.

### Baf A1 blocks virus-cell fusion

Baf A1 is thought to prevent endosome acidification, thereby preventing viral fusion and protein activation. Therefore, we tracked viral capsid and envelope proteins separately to visualize their fate during cell entry. To do this, we examined co-localization of these two viral components with markers of different parts of the endosomal hub (Rab7, Rab11, and LAMP1) in Baf A1-treated cells at 5–20 min p.i. (Additional file 3: Figure S3 and Additional file 4: Figure S4).

**Fig. 9:**
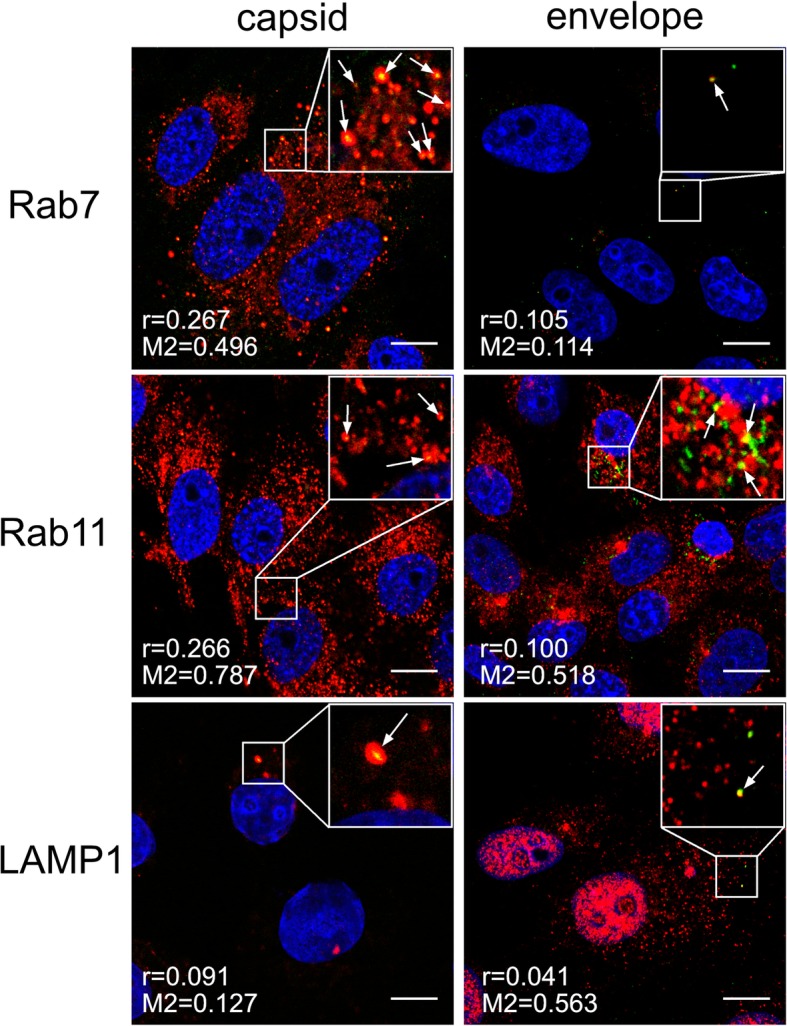
Baf A1 blocks virus-cell fusion. Co-localization between ZIKV and subcellular marker proteins in Baf A1-treated Vero cells 15 min p.i. Confocal images of 100 nM Baf A1-treated ZIKV-infected Vero cells presenting co-localization between ZIKV capsid or envelope proteins and Rab7, Rab11 and LAMP1 at 15 min p.i.. Rab7 – late endosomes marker protein, Rab11 – slow recycling endosomes marker protein, LAMP1 – lysosomes marker protein. ZIKV proteins are visualized in green, cellular proteins in red and nuclei are shown in blue. Co-localization coefficients for the representative presentations are indicated in the bottom left corners of the respective images; r – Pearson’s coefficient; M2 - Manders’ coefficient M2 (the virus overlapping with Rab7/Rab11/LAMP1). Scale bar = 10 μm

As described above, in non-treated cells the ZIKV capsid and envelope proteins travelled together to late endosomes, where fusion occurs. While capsid proteins entered the cytoplasm, envelope proteins were slowly re-traffic to the cell surface. In the presence of Baf A1, fusion was blocked, and both components tended to co-localize with Rab11- and LAMP-1-positive structures at 15 min p.i. (Fig. 9), suggesting that in the presence of Baf A1 viral particles do not fuse with the membrane of the vesicle. Rather, they are destined to undergo degradation in lysosomes. However, some are transported back to the cell surface via the slow recycling pathway.

### NH_4_Cl hampers infection inducing fast recycling of virions back to the cell surface

**Fig. 10:**
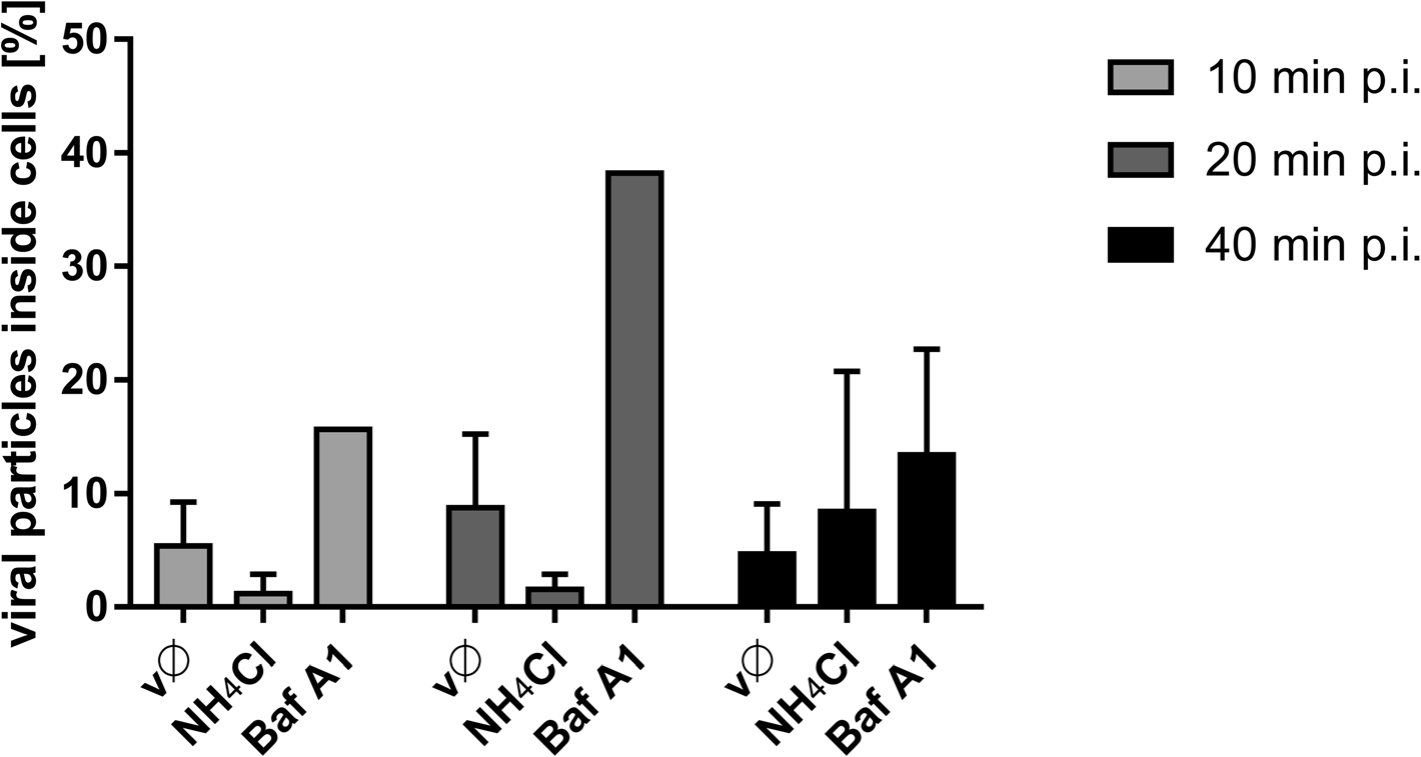
Intracellular ZIKV virions in cells treated with Baf A1 or NH_4_Cl. Ratio of ZIKV particles present inside cells and total number of ZIKV particles, assessed from confocal images of Vero cells infected with ZIKV in the absence (vØ) or presence of 100 nM Baf A1 (Baf A1) or 50 mM NH_4_Cl (NH_4_Cl). The data is presented as the mean ± SD

**Fig. 11:**
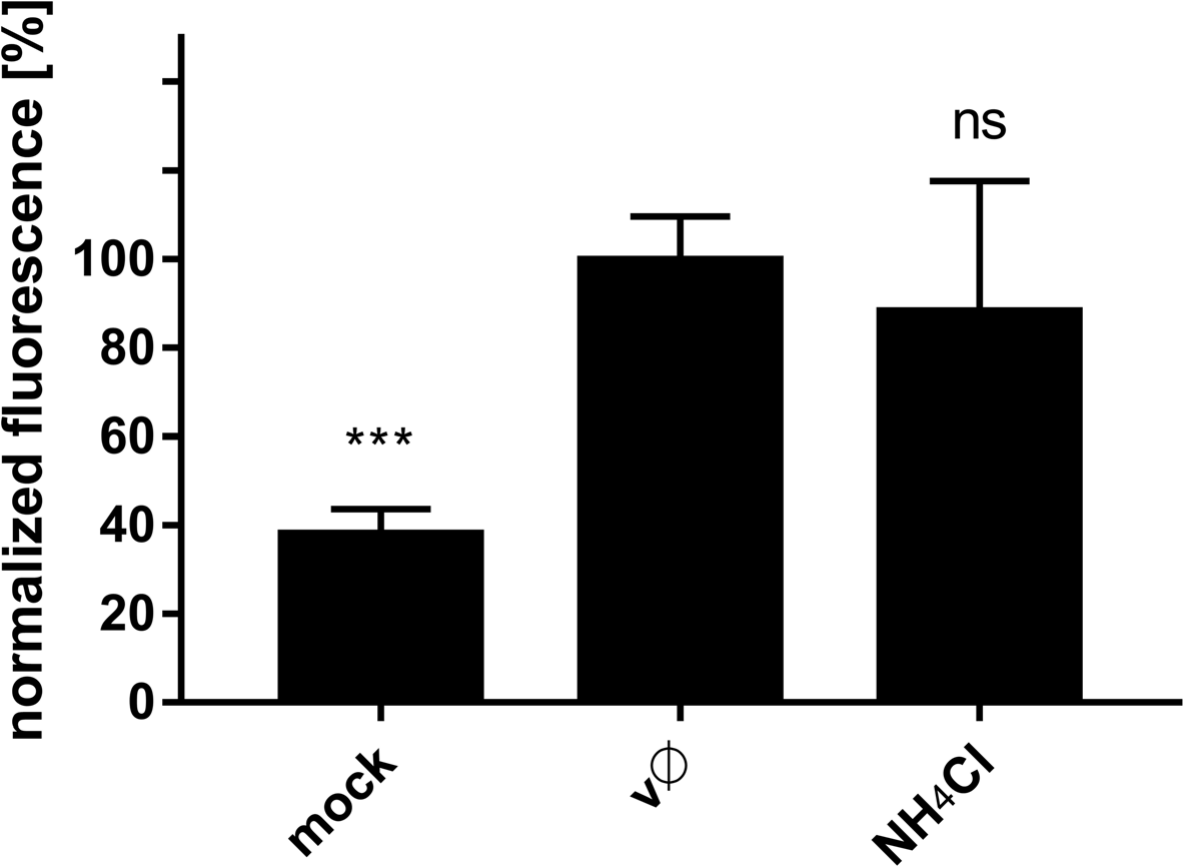
Influence of NH_4_Cl on ZIKV adhesion to the host cells. Flow cytometry analysis of viral adhesion to cells in the presence of NH_4_Cl was carried out. Vero cells pre-treated with 50 mM NH_4_Cl were overlaid with ZIKV stock and following 2 h incubation at 4 °C, they were fixed and ZIKV was immunostained with Alexa Fluor 488. Graph shows median fluorescence normalized to control, which corresponds to the proportion of ZIKV-positive cells in total cells population. vØ – inhibitor-untreated cells overlaid with ZIKV; NH_4_Cl –NH_4_Cl-treated cells overlaid with ZIKV; mock – mock-overlaid inhibitor-untreated cells. The data is presented as mean ± SD. To determine the significance of differences between compared groups, single-factor analysis of variance (ANOVA) was applied. *P* values < 0.05 were considered significant. One asterisk (*) identifies adjusted P values between 0.01 and 0.05, two asterisks (**) identify adjusted P values between 0.01 and 0.001, three asterisks (***) identify adjusted P values between 0.001 and 0.0001

A very different confocal image was observed when cells were treated with NH_4_Cl. In this case, few virus particles were observed in the cytoplasm (in contrast to Baf A1-treated cells, in which the number of internalized virions was similar to that in control cells) (Fig. 10). First, we used flow cytometry analysis to test whether NH_4_Cl affects binding of ZIKV to the cell surface, which would explain this phenomenon. As shown in Fig. 11, NH_4_Cl had no significant impact on ZIKV adhesion to cells.

**Fig. 12:**
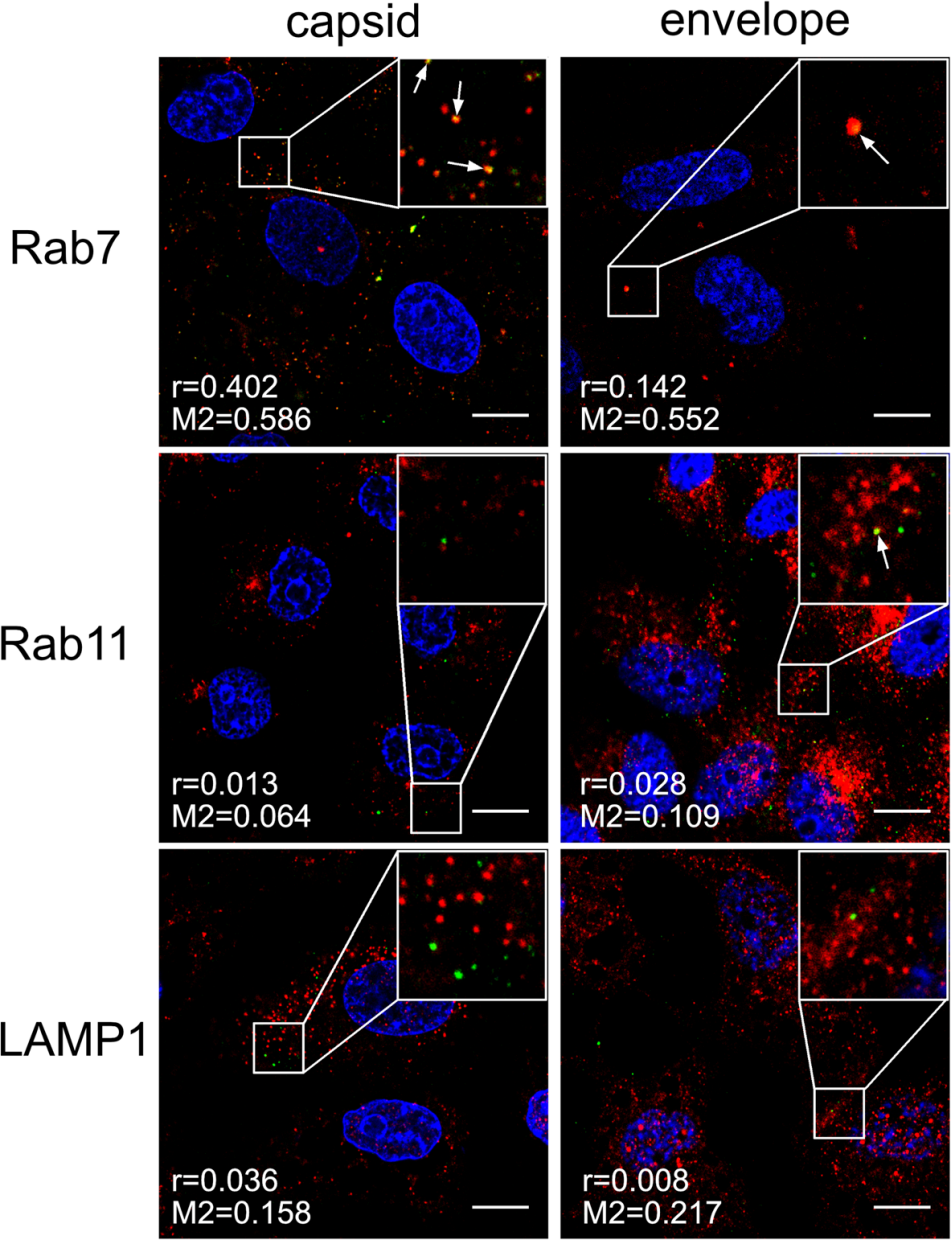
Co-localization between ZIKV and subcellular marker proteins in NH_4_Cl-treated Vero cells 20 min p.i. Confocal images of 50 mM NH_4_Cl ZIKV-infected Vero cells presenting co-localization between ZIKV capsid or envelope protein and Rab7, Rab11 and LAMP1 at 20 min p.i.. Rab7 – late endosomes marker protein, Rab11 – slow recycling endosomes marker protein, LAMP1 – lysosomes marker protein. ZIKV proteins are visualized in green, cellular proteins in red and nuclei are shown in blue. Co-localization coefficients for the representative presentations are indicated in the bottom left corners of the respective images; r – Pearson’s coefficient; M2 - Manders’ coefficient M2 (the virus overlapping with Rab7/Rab11/LAMP1). Scale bar = 10 μm

**Fig. 13:**
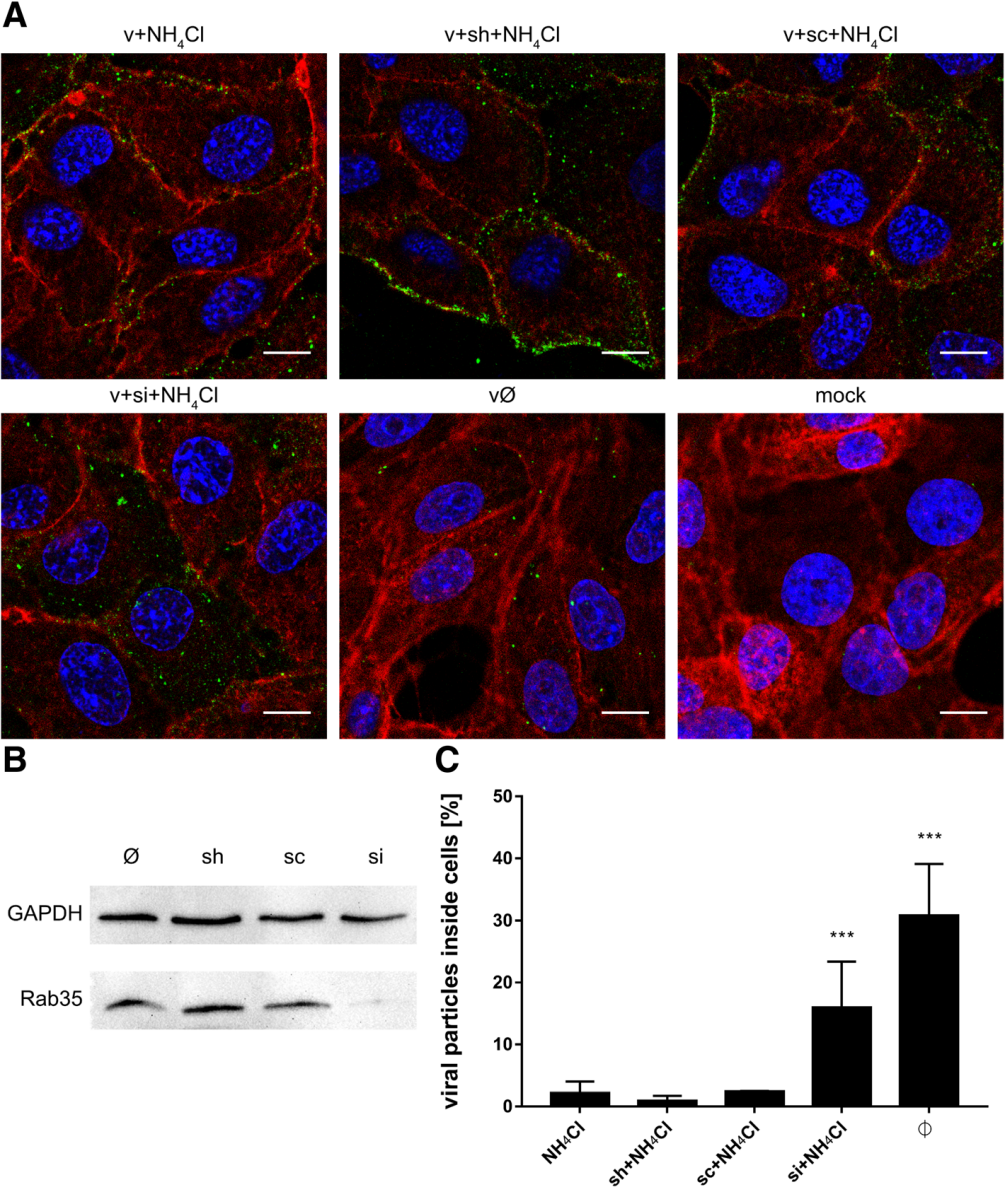
Subcellular localization of ZIKV in Rab35-depleted cells upon NH_4_Cl treatment. (**a**) Confocal analysis of ZIKV localization 1 h p.i. in NH_4_Cl-treated Vero cells. ZIKV envelope protein is visualized in green, actin cytoskeleton stained in red to show the cell boundaries and nuclei are shown in blue. Scale bar = 10 μm. (**b**) Western blot analysis of the efficiency of siRNA-dependent Rab35 silencing (Rab35 expression in Vero cells compared to GAPDH expression in these cells). M – BlueStar prestained protein marker; Ø – normal non-transfected Vero cells. (**c**) Graph representing the percent of ZIKV particles present inside cells related to the total number of ZIKV particles, assessed from confocal images of siRNA-transfected and all control Vero cells infected with ZIKV H/PF/2013 in the presence of 50 mM NH_4_Cl. v + NH_4_Cl - ZIKV-infected, NH_4_Cl-treated, non-transfected cells; v + sh + NH_4_Cl - ZIKV-infected, NH_4_Cl-treated, sham transfected cells; v + sc + NH_4_Cl - ZIKV-infected, NH_4_Cl-treated, scrambled siRNA transfected cells; v + si + NH_4_Cl - ZIKV-infected, NH_4_Cl-treated, Rab35-specific siRNA transfected cells; vØ - ZIKV-infected, NH_4_Cl-untreated, non-transfected cells; mock - mock-infected, NH_4_Cl-untreated, non-transfected cells. The data is presented as the mean ± SD. To determine the significance of differences between compared groups, single-factor analysis of variance (ANOVA) was applied. P values < 0.05 were considered significant. One asterisk (*) identifies adjusted P values between 0.01 and 0.05, two asterisks (**) identify adjusted P values between 0.01 and 0.001, three asterisks (***) identify adjusted P values between 0.001 and 0.0001

Next, despite the low number of internalized viral particles, we examined their co-localization with cellular markers (Additional file 5: Figure S5 and Additional file 6: Figure S6). In NH_4_Cl-treated cells, both the capsid and envelope proteins co-localized with Rab7 (Fig. 12), suggesting that internalized virions follow their normal entry route. Moreover, slightly increased co-localization of Rab11 with the envelope protein, but not the capsid protein, at 20 min p.i. (Fig. 12), may suggest that, at least for these single virus particles, fusion actually occurs. We observed that, in the presence of NH_4_Cl, although the virus may enter the cell, the number of internalized viruses was very small. Because the inhibitor did not affect the virus-cell interaction, we hypothesized that the observed phenomenon may result from extensive anterograde transport. We did not observe co-localization with Rab27; therefore, we excluded the role of exosomes in NH_4_Cl-redirected virus trafficking (data not shown). To verify the role of fast recycling endosomes, we used a different approach. Because it was very difficult to visualize this rapid process, we examined subcellular localization of ZIKV upon NH_4_Cl treatment of Vero cells in which expression of Rab35, a marker protein that guides endosomes to the fast recycling track, was transiently silenced. In the presence of NH_4_Cl, the majority of virions localized to the cell surface of control and scrambled siRNA-transfected cells, whereas those in cells transfected with Rab35-specific siRNA were retained within the cell (trapped near the cell surface) until 1 h p.i. (Fig. 13). This observation led us to conclude that NH_4_Cl impairs ZIKV infection at an early stage by redirecting virions back to the cell surface.

### NH_4_Cl-induced re-modelling of intracellular trafficking: effects on viral replication, assembly, and egress

**Fig. 14:**
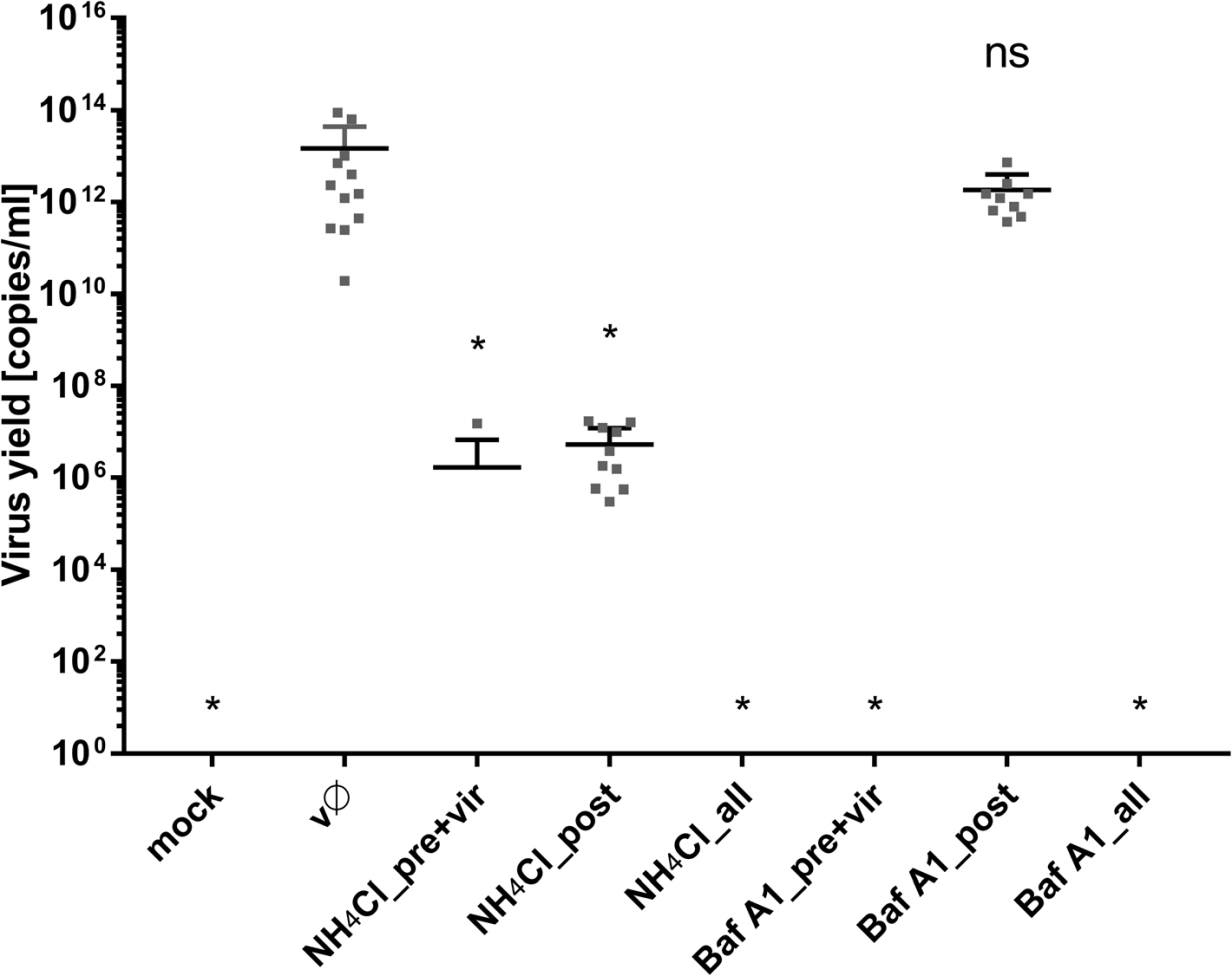
NH_4_Cl interfere with late stages of ZIKV infection. Vero cells were treated Baf A1 and NH_4_Cl in 3 settings: (pre + vir) prior to + during ZIKV infection (t = 1 h + 2 h); (all) prior to, during and post-ZIKV infection (t = 1 h + 2 h + 72 h) or (post) post-ZIKV infection only (t = 72 h, starting from the time point 2 h p.i.). 3 days p.i. viral RNA was isolated from medium and quantified with RT-qPCR. mock – mock infected cells; vØ – ZIKV- infected cells; NH_4_Cl – 50 mM NH_4_Cl; BafA1–100 nM bafilomycin A1; control – inhibitor-untreated, non-infected cells. To determine the significance of differences between compared groups, single-factor analysis of variance (ANOVA) was applied. P values < 0.05 were considered significant. One asterisk (*) identifies adjusted P values between 0.01 and 0.05, two asterisks (**) identify adjusted P values between 0.01 and 0.001, three asterisks (***) identify adjusted P values between 0.001 and 0.0001

Intracellular trafficking is important for virus replication, not only at the early stages but also during virus assembly and egress. The data regarding NH_4_Cl-mediated remodeling of the endosomal hub led us to hypothesize that the compound may also interfere with the late stages of infection. To confirm this, Vero cells were infected for 2 h with ZIKV under normal conditions (i.e., in the absence of pH-modifying agents). Afterwards, cells were rinsed thrice with PBS and then incubated at 37 °C for 3 days in standard medium containing Baf A1 or NH_4_Cl. Although Baf A1 did not affect the viral yield, NH_4_Cl led to a 6.5 log reduction (Fig. 14), highlighting differences in the mode of action between these two agents and identifying a role for NH_4_Cl during the late stages of infection.

## Discussion

During infection, viruses hijack the inward transport machinery of the cell [42–45]. While some viruses are able to fuse with the cell membrane and initiate infection almost immediately after entry, others need to be ferried long distances, e.g., during infection of neural cells [46–49]. This study focused on events that occur after the initial interaction between ZIKV and its cellular receptor.

As observed for other flaviviruses and for ZIKV in different in vitro models [10, 12, 14], our findings demonstrate that ZIKV enters Vero cells by clathrin-dependent endocytosis. Co-localization of viral particles with clathrin was observed 2–10 min p.i., as expected for CME. However, synchronization of virus entry was not ideal due to the fact that the process was regulated by temperature; the numerical co-localization rates were significant but moderate (Fig. 1; Pearson’s coefficient ranging from 0.103 to 0.216 and Manders’ coefficients up to 0.384). To ensure that the observed co-localization is not an artifact, complementary studies, including clathrin silencing (Fig. 2) or CME-specific inhibitors (Fig. 3), were carried out and confirmed our initial observations.

Subsequent to clathrin-dependent internalization, the virus is encapsulated within the endosome prior to delivery to a precisely defined location. During transport, the microenvironment within the maturing endosome changes gradually; the falling pH, cellular proteases, alterations in the vesicle’s membrane content, and fluctuations in redox potential affect the cargo. Viruses are fine-tuned to become active only under conditions that maximize the chances of a productive infection; therefore in most cases this event takes place at a precisely defined site within the endosomal hub [50–58]. Tracking single dengue virus particles revealed that they pass the early endosomes and fuse predominantly with vesicle membranes as they mature into late endosomes at 10–13 min p.i. [42]. Our observations of ZIKV trafficking are congruent with these findings; we noticed separation of the ZIKV envelope and capsid protein trafficking routes between 10 and 15 min p.i. These two viral proteins were seen together for the last time in Rab7-positive structures. These results are also consistent with the data obtained with a novel a novel surrogate-receptor approach described by Rawle et al. [59]. Later the ZIKV envelope appeared to be transported back to the cell surface via slow recycling endosomes. The latter observation is striking, because it is commonly believed that, after endocytosis, viruses avoid leaving evidence of their presence on the plasma membrane as this delays detection by immunosurveillance system [46].

Multiple studies on flaviviruses showed that to acquire fusion competence the envelope proteins need to be primed proteolytically at two sites [50, 60–62]. First cleavage occurs during transport through the *trans*-Golgi network, where a tight complex of prM and E proteins on the surface of newly formed immature virion undergoes a low pH-induced conformational change, followed by cleavage by furin or a furin-like protease [60, 61]. Consequently, mature infectious virions that carry the dimeric E protein in a metastable conformation are released from the cell [62]. The second event occurs during endocytosis into a permissive cell; when the E protein is exposed to low pH, it undergoes rearrangment and enters a fusion-competent state [50].

To identify the factors that activate the ZIKV fusion protein, we used two classes of protease inhibitors. Inactivation of cysteine proteases (e.g., cathepsins) and serine proteases did not affect the infection process. However, furin inhibitors hampered the replication cycle, especially when present during the late stages. This observation is consistent with a common belief that furin in essential for maturation of ZIKV, similarly as for other flaviviruses. Despite the fact that ZIKV E and M proteins structures have been resolved [63, 64] and furin-specific cleavage site in ZIKV sequences is present [65], no report showing the role of furin during ZIKV replication is available (Fig. 5) [60, 65–72].

One may conclude that furin or a furin-like enzyme activates progeny viruses, while no second protease is required during entry to susceptible cell (as reported for other members of *Flaviviridae* family) [60, 61]. By contrast, some results advocating a role for furin during virus maturation may be due to an artifact, linked to the low specificity of the protease inhibitors used [73].

Here, we show that entry of ZIKV depends on the endosomal pH, and that endosome acidification is a prerequisite for fusion. Because we wanted to map the entry of a single virion into the cell, we used two agents commonly used to assess virus dependence on acidic environments. NH_4_Cl is a water-soluble salt of ammonia that diffuses into the endosome and acts as a proton sink, thereby inhibiting acidification of the endosome [74]. The second compound, Baf A1, is a vacuolar type H^+^-ATPase inhibitor that binds to the V0 sector subunit c of the ATPase complex and inhibits H^+^ translocation, thereby blocking endosome acidification [75]. Even though the outcome of both treatments was similar, the mechanism of action appeared to be very different.

In the presence of Baf A1, ZIKV particles were internalized and trafficked to the late endosomal compartment. However, due to altered pH in the vesicles, virions were not able to enter a fusogenic state and remained trapped in the endosomes, which seemed to progress slowly to lysosomes. Partial degradation and, to a lesser extent, slow recycling to the cell surface are probably responsible for removal of the degradation products, so that whole virions share the fate of the envelope protein. By contrast, in the presence of NH_4_Cl the majority of virions are retained on the cell surface, which suggests limited virus attachment/entry. First, we confirmed that the virus–receptor interaction was not affected by the basic compound; no alterations were observed. Consequently, potential causes of such a phenomenon include limited internalization or re-trafficking to the cell surface. A limited number of virions localizing inside the cells meant the we could not obtain credible results from co-localization assays. Because no significant increase in co-localization with the slow recycling endosome marker Rab11 was observed in NH_4_Cl-treated cells, we tried to inhibit the fast recycling machinery by transiently transfecting Vero cells with siRNAs targeting the fast recycling endosome marker Rab35. Depleting Rab35 resulted in retention of virions inside the cell, showing that the NH_4_Cl-mediated inhibition results from rewiring of the endosomal hub rather than a simple increase in endosomal pH.

## Conclusions

To summarize, we mapped the entry route of ZIKV using Vero cells as the research model. The results are consistent with data on ZIKV entry into other cell types including primary cells, suggesting that the virus uses a universal entry mechanism. First, the virus uses CME to enter the cell, and then travels through the endosomal compartment to reach the late endosomes prior to fusion. Subsequently, the viral envelope tends to recycle to the cell surface. While it is essential that progeny viruses are primed by furin-like enzymes during assembly, entry seems to be protease-independent. Interestingly, we noted that NH_4_Cl (believed to simply buffer the endosomal microenvironment) is in fact re-directing the cargo and ejecting it into the extracellular space. In our previous study we reported similar example of virus entry pathway redirection [76], showing that external factors may affect virus entry in an non-obvious way. We believe that the results presented herein not only increase our understanding of ZIKV biology, but also provide novel molecular targets for future therapies.

## Additional files

Additional file 1: Confocal images of ZIKV-infected Vero cells presenting co localization between ZIKV structural proteins and Rab7, Rab11 and LAMP1 at indicated time points p.i.. Rab7 – late endosomes marker protein, Rab11 – slow recycling endosomes marker protein, LAMP1 – lysosomes marker protein. ZIKV capsid and envelope proteins are visualized in green, cellular proteins are shown in red and nuclei in blue. Co‑localization coefficients indicated in the bottom left corners of the images are presented as mean ± SD of at least two independent experiments; r – Pearson’s coefficient; M2 - Manders' coefficient M2 (ZIKV capsid/envelope protein overlapping with Rab7/Rab11/LAMP1). Scale bar = 10 μm. **Figure S1.** Co-localization profile for ZIKV capsid protein and subcellular marker proteins in Vero cells. (TIFF 2158 kb)

Additional file 2: **Figure S2.** Co-localization profile for ZIKV envelope protein and subcellular marker proteins in Vero cell. (TIFF 2315 kb)

Additional file 3: **Figure S3.** Co-localization profile for ZIKV capsid protein and subcellular marker proteins in Baf A1-treated Vero cells. (TIFF 2207 kb)

Additional file 4: **Figure S4.** Co-localization profile for ZIKV envelope protein and subcellular marker proteins in Baf A1-treated Vero cells. (TIFF 1894 kb)

Additional file 5: **Figure S5.** Co-localization profile for ZIKV capsid protein and subcellular marker proteins in NH4Cl-treated Vero cells. (TIFF 2103 kb)

Additional file 6: **Figure S6.** Co-localization profile for ZIKV envelope protein and subcellular marker proteins in NH4Cl-treated Vero cells. (TIFF 1722 kb)

## Abbreviations

AMTD: Amantadine (1-adamantylaminean)
Baf A1: Bafilomycin A1 [(3Z,5E,7R,8S,9S,11E,13E,15S,16R)-8-hydroxy-16-[(1S,2R,3S)-2-hydroxy-1-methyl-3-[(2R,4R,5S,6R)-tetrahydro-2,4-dihydroxy-5-methyl-6-(1-methylethyl)-2H-pyran-2-yl]butyl]-3,15-dimethoxy-5,7,9,11-tetramethyloxacyclohexadeca-3,5,11,13-tetraen-2-one)
Cam: Camostat [4-[[4-[(aminoiminomethyl)amino]benzoyl]oxy] benzeneacetic acid 2-(dimethylamino)-2-oxoethyl ester methanesulfonate)
CME: Clathrin-mediated endocytosis
CMK: Decanoyl-arg-val-lys-arg-chloromethylketone
Dyn: Dynasore [3-hydroxynaphthalene-2-carboxylic acid (3,4-dihydroxybenzylidene)hydrazide]
E64: Trans-epoxysuccinyl-L-leucylamido (4-guanidino) butane, L-trans-3-Carboxyoxiran-2-carbonyl-L-leucylagmatine, N-(trans-epoxysuccinyl)-L-leucine 4-guanidinobutylamide
EEA1: Early endosome antigen 1
MM: MitMAB (tetradecyltrimethylammonium bromide)
PtS: PitStop 2 [N-[5-[4-bromobenzylidene]-4-oxo-4,5-dihydro-1,3-thiazol-2-yl] naphthalene-1-sulfonamide]
ZIKV: Zika virus
