# Supplementary figures and images for: Zika virus: mapping and reprogramming the entry
Source: Cell Commun Signal. 2019 May 3;17:41. doi: 10.1186/s12964-019-0349-z (PMC6500006; doi:10.1186/s12964-019-0349-z)

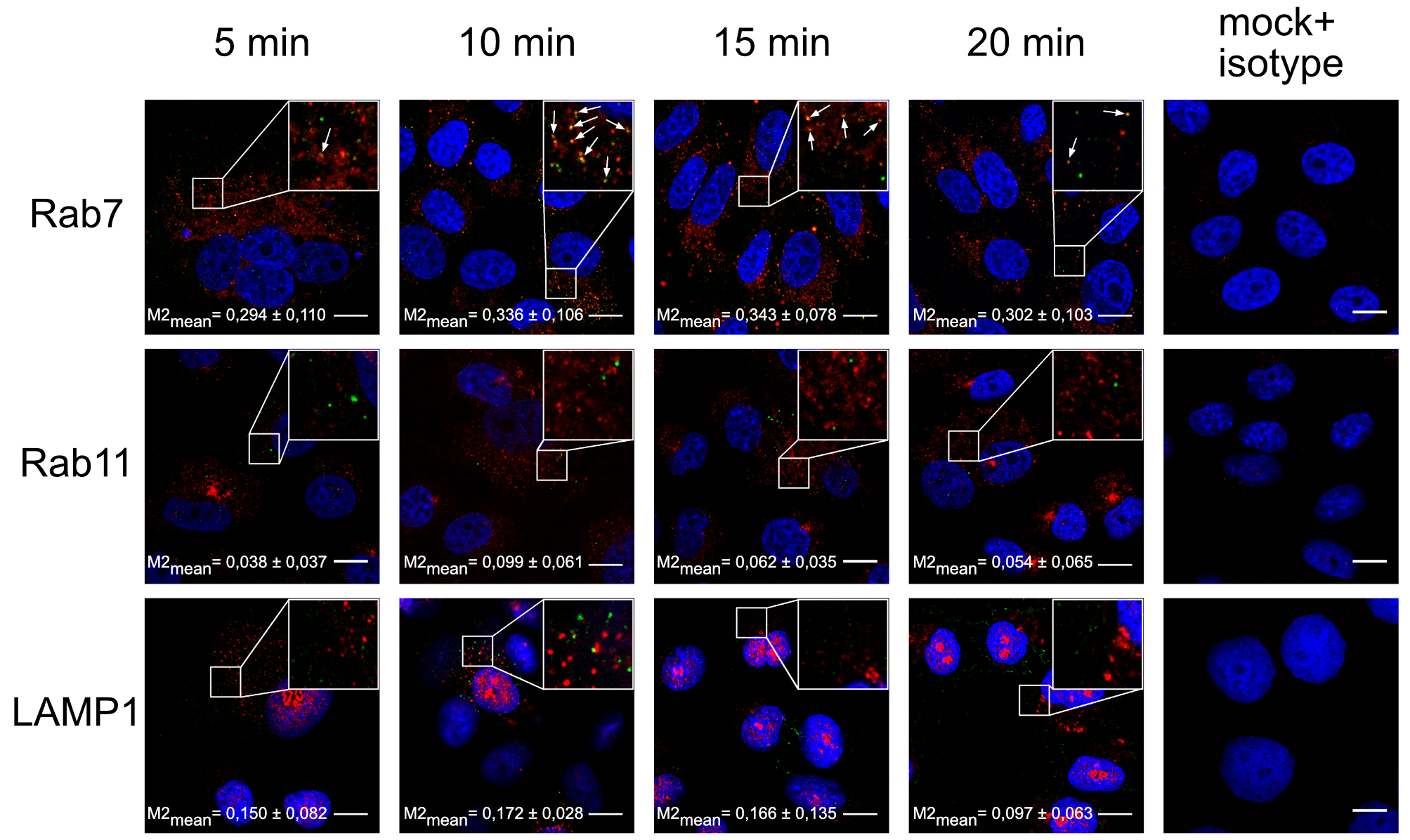

Supplement: Supplementary file 1 — Confocal images of ZIKV-infected Vero cells presenting co localization between ZIKV structural proteins and Rab7, Rab11 and LAMP1 at indicated time points p.i.. Rab7 – late endosomes marker protein, Rab11 – slow recycling endosomes marker protein, LAMP1 – lysosomes marker protein. ZIKV capsid and envelope proteins are visualized in green, cellular proteins are shown in red and nuclei in blue. Co‑localization coefficients indicated in the bottom left corners of the images are presented as mean ± SD of at least two independent experiments; r – Pearson’s coefficient; M2 - Manders' coefficient M2 (ZIKV capsid/envelope protein overlapping with Rab7/Rab11/LAMP1). Scale bar = 10 μm. Figure S1. Co-localization profile for ZIKV capsid protein and subcellular marker proteins in Vero cells. (TIFF 2158 kb) [file 12964_2019_349_MOESM1_ESM.tiff]

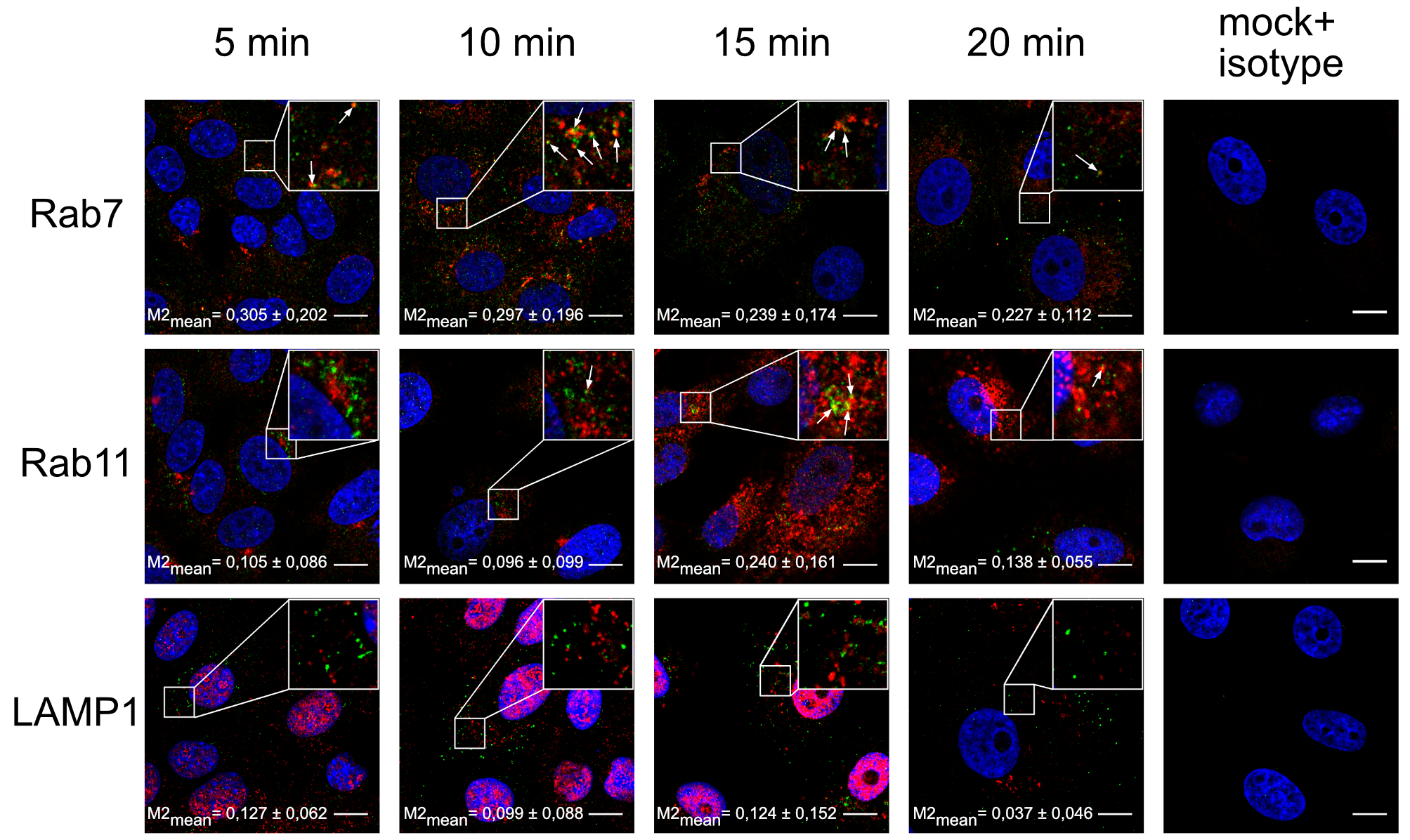

Supplement: Supplementary file 2 — Figure S2. Co-localization profile for ZIKV envelope protein and subcellular marker proteins in Vero cell. (TIFF 2315 kb) [file 12964_2019_349_MOESM2_ESM.tiff]

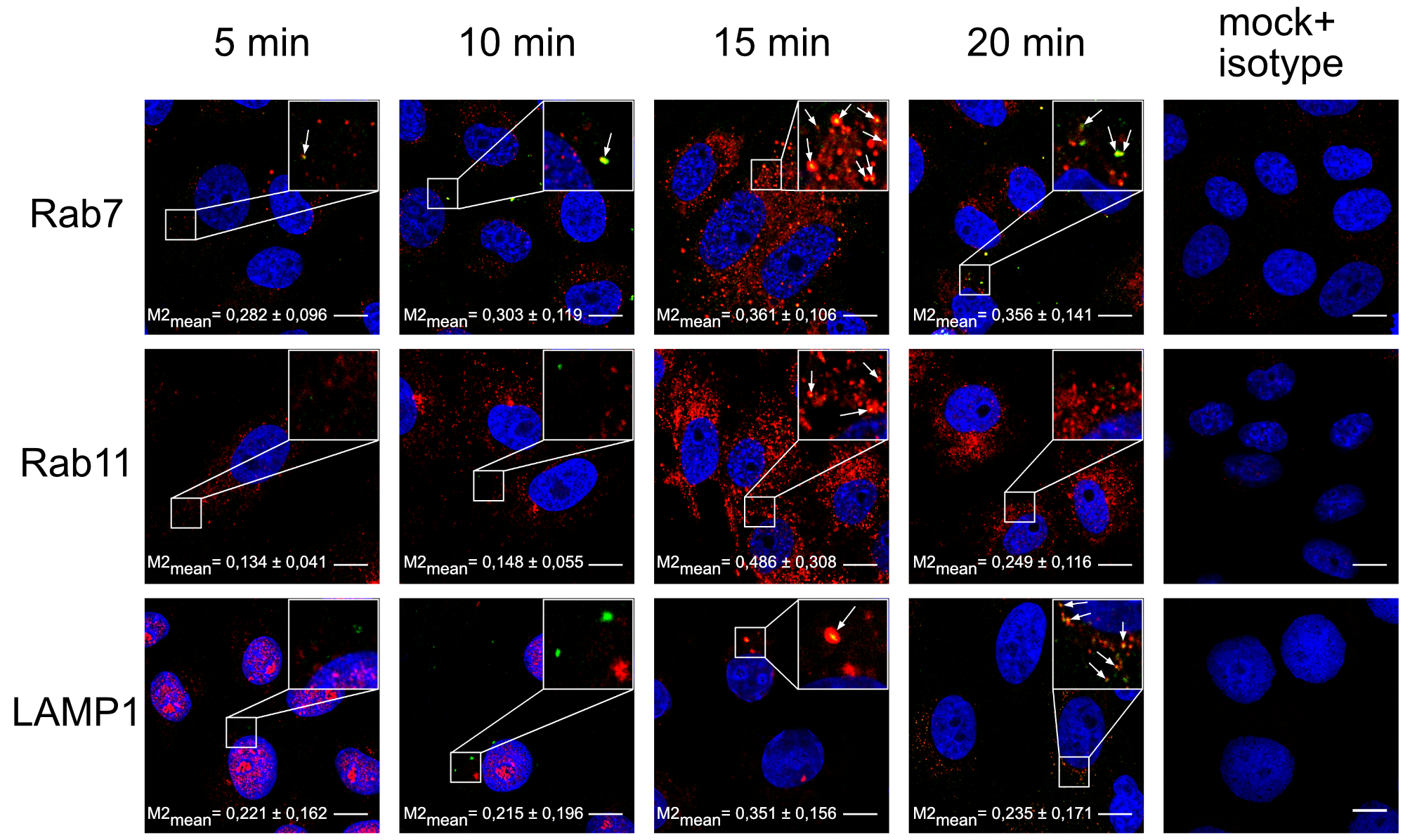

Supplement: Supplementary file 3 — Figure S3. Co-localization profile for ZIKV capsid protein and subcellular marker proteins in Baf A1-treated Vero cells. (TIFF 2207 kb) [file 12964_2019_349_MOESM3_ESM.tiff]

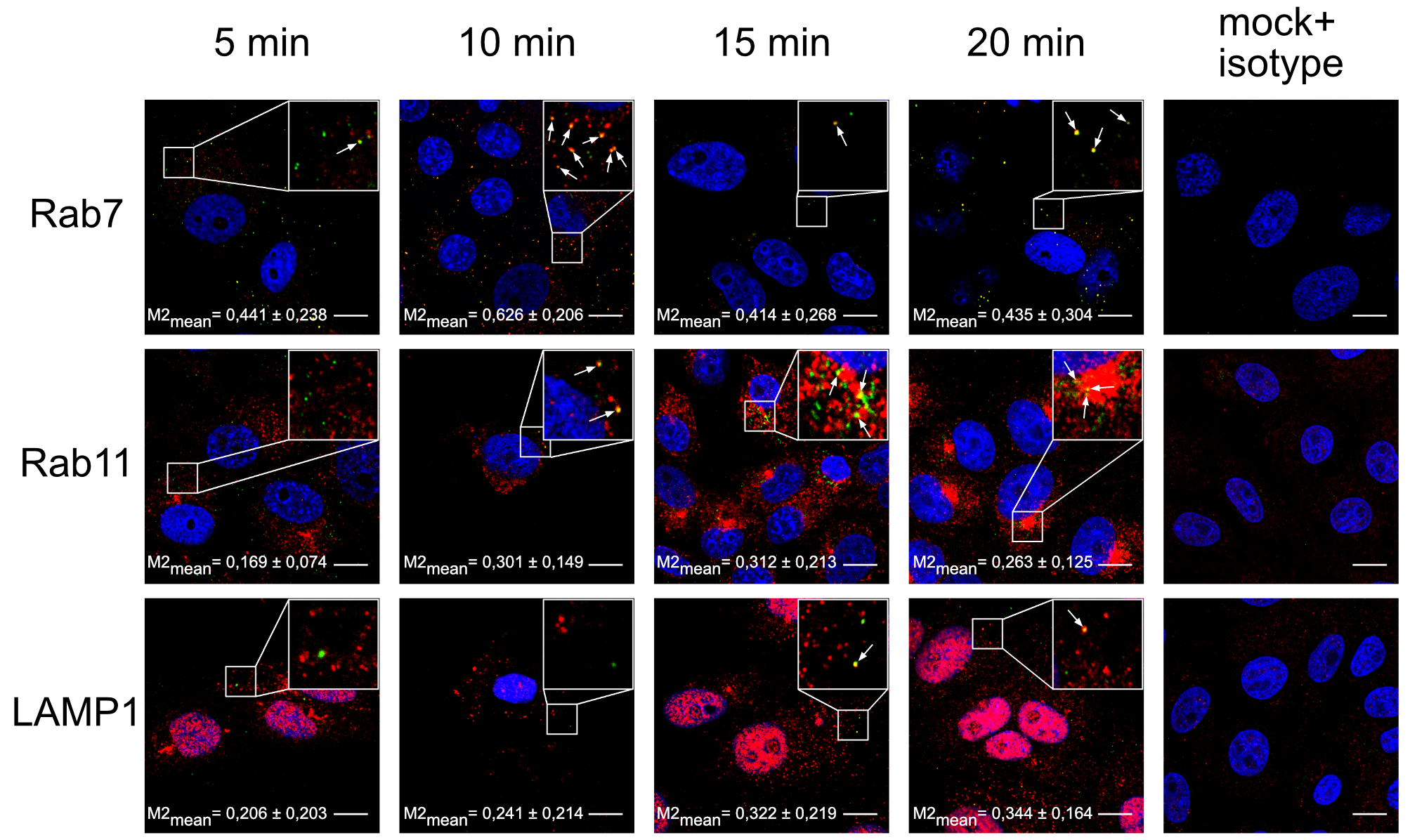

Supplement: Supplementary file 4 — Figure S4. Co-localization profile for ZIKV envelope protein and subcellular marker proteins in Baf A1-treated Vero cells. (TIFF 1894 kb) [file 12964_2019_349_MOESM4_ESM.tiff]

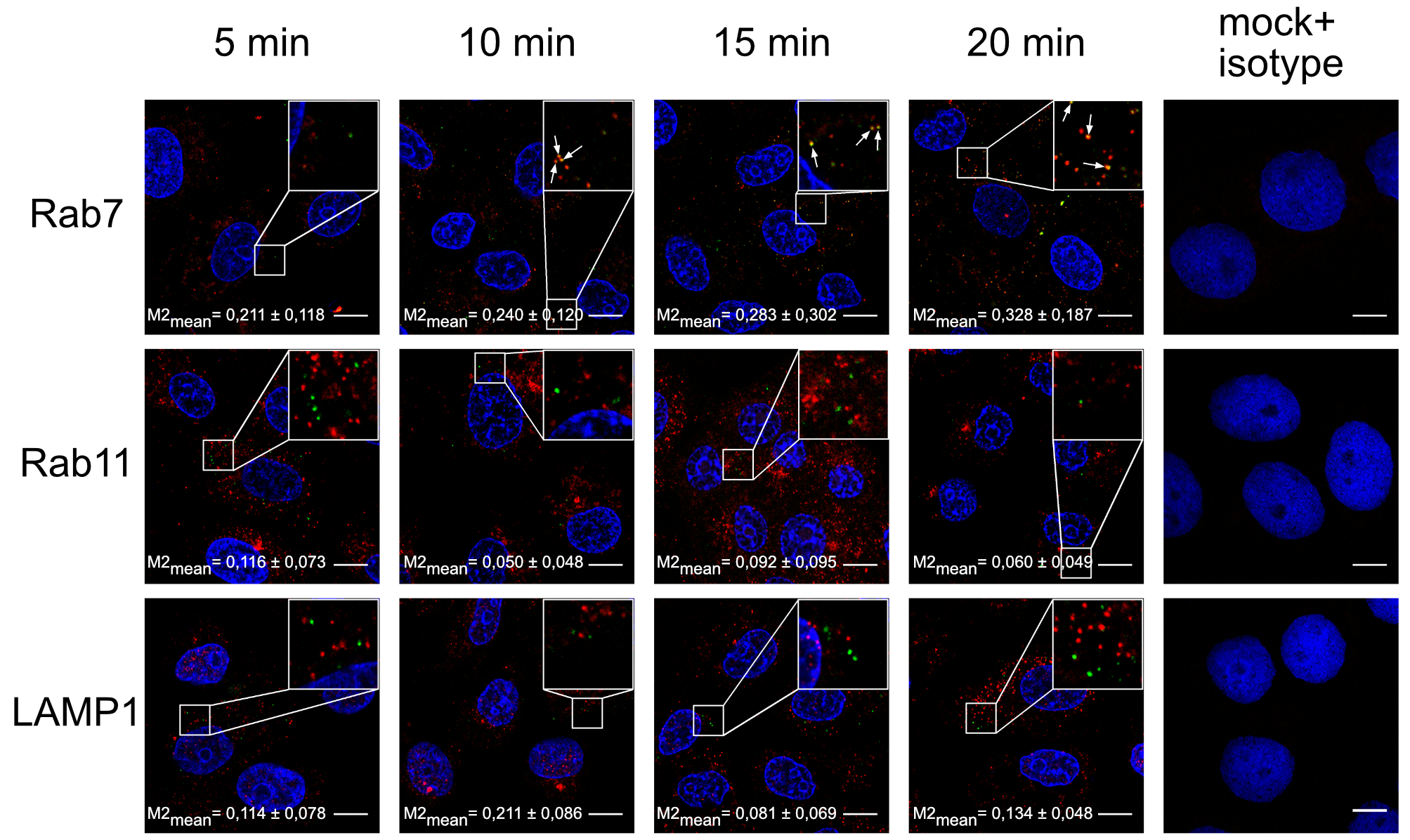

Supplement: Supplementary file 5 — Figure S5. Co-localization profile for ZIKV capsid protein and subcellular marker proteins in NH4Cl-treated Vero cells. (TIFF 2103 kb) [file 12964_2019_349_MOESM5_ESM.tiff]

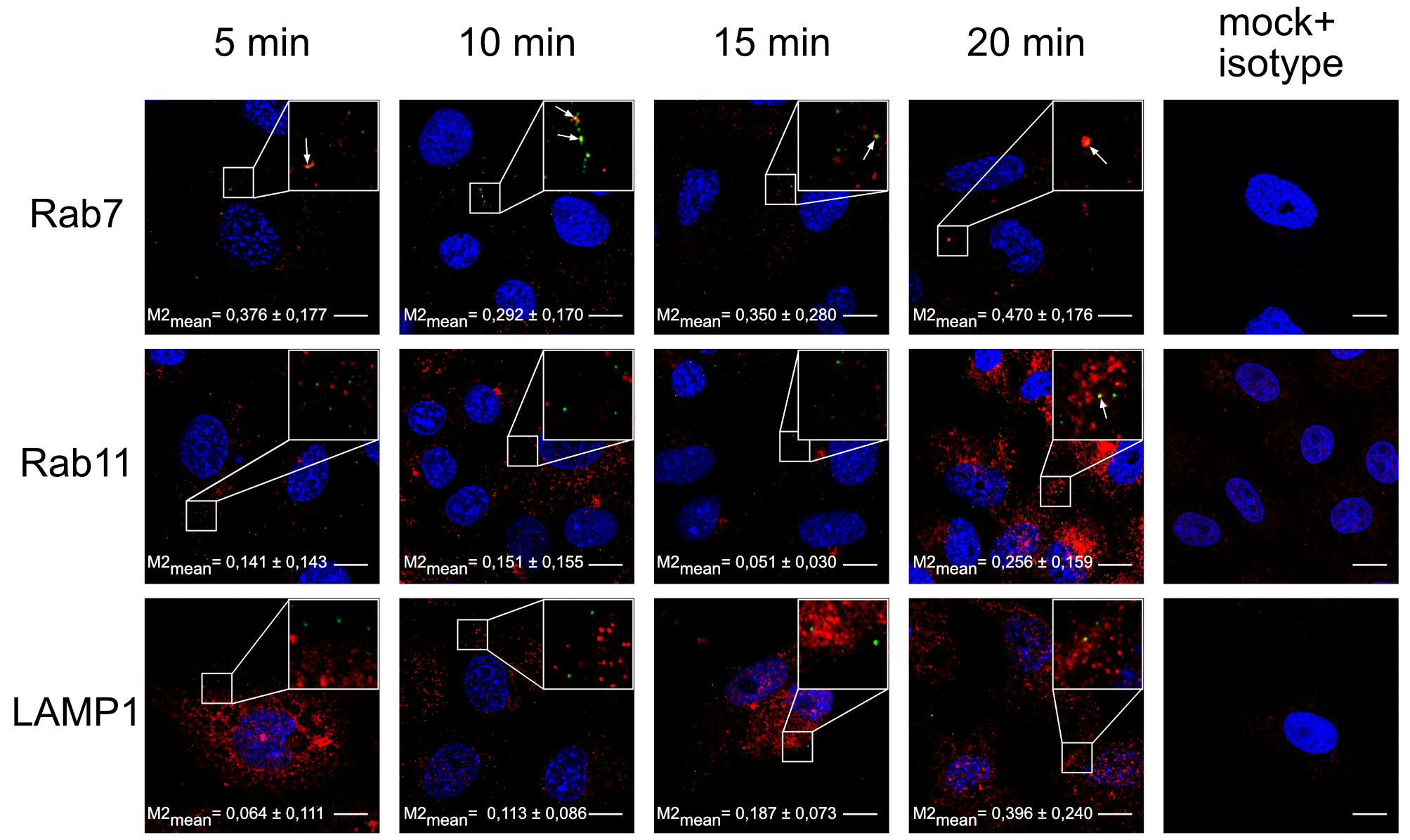

Supplement: Supplementary file 6 — Figure S6. Co-localization profile for ZIKV envelope protein and subcellular marker proteins in NH4Cl-treated Vero cells. (TIFF 1722 kb) [file 12964_2019_349_MOESM6_ESM.tiff]
